# Emerging Iontronic Sensing: Materials, Mechanisms, and Applications

**DOI:** 10.34133/2022/9867378

**Published:** 2022-08-14

**Authors:** Yao Xiong, Jing Han, Yifei Wang, Zhong Lin Wang, Qijun Sun

**Affiliations:** ^1^Beijing Institute of Nanoenergy and Nanosystems, Chinese Academy of Sciences, Beijing 101400, China; ^2^School of Nanoscience and Technology, University of Chinese Academy of Sciences, Beijing 100049, China; ^3^School of Materials Science and Engineering, Georgia Institute of Technology, Atlanta GA 30332, USA; ^4^Center on Nanoenergy Research, School of Physical Science and Technology, Guangxi University, Nanning 530004, China

## Abstract

Iontronic sensors represent a novel class of soft electronics which not only replicate the biomimetic structures and perception functions of human skin but also simulate the mechanical sensing mechanism. Relying on the similar mechanism with skin perception, the iontronic sensors can achieve ion migration/redistribution in response to external stimuli, promising iontronic sensing to establish more intelligent sensing interface for human-robotic interaction. Here, a comprehensive review on advanced technologies and diversified applications for the exploitation of iontronic sensors toward ionic skins and artificial intelligence is provided. By virtue of the excellent stretchability, high transparency, ultrahigh sensitivity, and mechanical conformality, numerous attempts have been made to explore various novel ionic materials to fabricate iontronic sensors with skin-like perceptive properties, such as self-healing and multimodal sensing. Moreover, to achieve multifunctional artificial skins and intelligent devices, various mechanisms based on iontronics have been investigated to satisfy multiple functions and human interactive experiences. Benefiting from the unique material property, diverse sensing mechanisms, and elaborate device structure, iontronic sensors have demonstrated a variety of applications toward ionic skins and artificial intelligence.

## 1. Introduction

Human skin is capable to perceive temperature, humidity, pressure, and different types of stimuli and conditions [1–7]. Mimicry of the complex sensory characteristics of skin sensing through electronic methods is a quite fascinating research direction for future development of prosthetic skin, human-machine interactive electronics, and artificial intelligence. Despite significant progresses in electronic skin (E-skin) sensors consisting of electronic counterparts (e.g., conductors, semiconductors, and dielectrics) have been achieved, there remain profound challenges in skin-like touch sensation which requires the artificial skin to exhibit mechanical flexibility/stretchability, high-resolution sensing, and prompt feedback to external stimuli.

For stretchable electronics, one effective approach pioneered by Bao's group is to implement system integration with intrinsically stretchable materials, e.g., graphene [8, 9], carbon nanotubes (CNTs) [10, 11], silver (Ag) nanowires [12], conductive polymers [13], and metal meshes [14, 15, 16–19]. Another mainstream approach to realize the stretchability of electronics by Rogers and Huang's groups [20–27] is emphasizing on ingenious design of electrode structures and geometries. Henceforth, the strategies of unique materials composition, ingenious structural design, and innovative combination of components have been broadly applied for diverse artificial skin devices.

Besides material innovation and structural design, introducing iontronic sensing has brought new vitality to biomimetic soft electronics as a disruptively evolutionary technology. As the iontronic devices adopt the same ion conduction as biological system, iontronic sensing can not only reconstruct the sensing topological structures of human skin but also emulate mechanical sensing mechanism relying on ion migration under environmental stimuli. Thus, the iontronic devices represent significant conceptual similarities with biological systems [28–30]. Relying on the similar mechanism of skin perception, the iontronic sensors can respond to mechanical stimuli through the migration and redistribution of ions. Compared to conventional E-skin devices, such similarity ensures the iontronic devices to simulate more advanced biocompatible interface and intelligent human interactive platforms. With the aid of advanced signal processing techniques and automatic control techniques, the iontronics can be significantly extended to multifunctional and intelligent applications.

**Figure 1:**
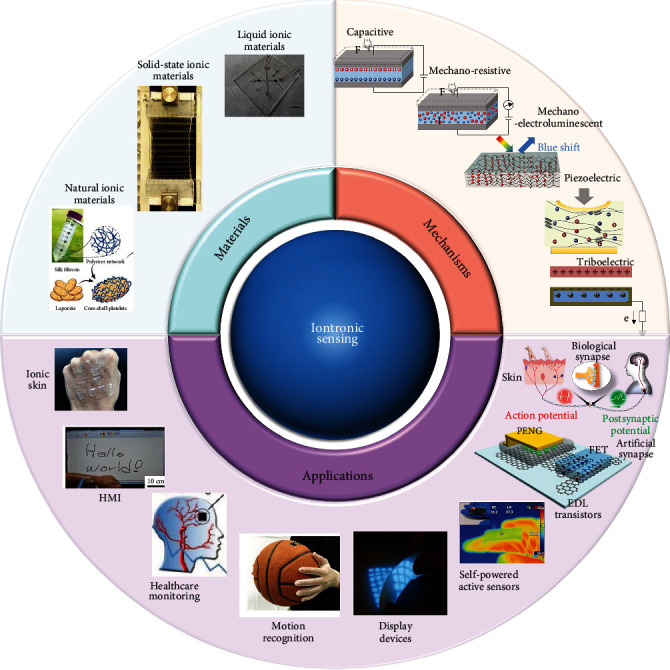
The conceptual schematics of iontronic sensing in terms of materials, mechanisms, and applications. Reproduced with permission [40], copyright 2014, Macmillan Publishers Limited. All rights reserved. Reproduced with permission [64], copyright 2012, Nature Publishing Group, a division of Macmillan Publishers Limited. Reproduced with permission [97], copyright 2019, The Authors. Published by WILEY-VCH Verlag GmbH & Co. KGaA, Weinheim. Reproduced with permission [107], copyright 2017, The Authors, some rights reserved; exclusive licensee American Association for the Advancement of Science. Reproduced with permission [105], copyright 2016, American Association for the Advancement of Science. Reproduced with permission [163], copyright 2017, WILEY-VCH Verlag GmbH & Co. KGaA, Weinheim. Reproduced with permission [109], copyright 2017, WILEY-VCH Verlag GmbH & Co. KGaA, Weinheim. Reproduced with permission [113], copyright 2016, American Association for the Advancement of Science. Reproduced with permission [169], copyright 2020, WILEY-VCH Verlag GmbH & Co. KGaA, Weinheim. Reproduced with permission [151], copyright 2015, WILEY-VCH Verlag GmbH & Co. KGaA, Weinheim. Reproduced with permission [177], copyright 2021, The Author(s).

The conceptual schematics of iontronic sensors in terms of materials, mechanisms, and applications are described in Figure 1. By virtue of excellent stretchability, high transparency, and mechanical conformality, Pan's group firstly reports on adopting ionic liquids to fabricate iontronic sensors with ultrahigh sensitivity [31]. Afterwards, intensive attempts have been made to explore various novel ionic materials (e.g., ionic gels and hydrogels) to devise iontronic sensors with skin-like perceptive properties. Besides, various working mechanisms based on iontronics allow for multifunctional artificial skins and intelligent devices, which can satisfy the additional wide-ranging industrial demands. The mechano-electroluminescent/electrochromic can endow the iontronics with the capability of quantitatively feeding back mechanical stimuli via electrical signals and visually mapping the distributed stimulations by luminescence and color change (which offers visible user interactive interfaces). Furthermore, the emerging piezoelectric and triboelectric technologies have dramatically promoted self-powered iontronic sensing system derived from biomechanical energy which is of great significance in solving the power supply problem for iontronic devices. Benefiting from the unique material property, diverse sensing mechanisms, and elaborate device structure, iontronic sensors have been demonstrated in various applications toward ionic skins and artificial intelligence.

In this review, we primarily elaborate the advanced technologies and versatile applications for the development of iontronic sensors toward ionic skins and artificial intelligence. We first introduce the recent progress in ionic materials (i.e., liquid ionic materials, solid-state ionic materials, and natural ionic materials) employed for the iontronic sensors with their intrinsic advantages (Section 2). We then summarize the working mechanisms based on iontronic sensors in Section 3, covering interfacial capacitive sensing, mechanoresistive sensing, mechano-electroluminescent/electrochromic sensing, piezoelectric sensing, triboelectric sensing, and other mechanisms originated from various integrated components. Based on different working mechanisms, we further summarize the recent progress of iontronic sensing devices in Section 4, including ionic skins and human interactive interfaces, wearable healthcare monitoring and motion recognition, visual interactive displays, self-powered active sensors, flexible electric-double-layer transistors, and artificial synapses. Finally, we review the challenges to iontronic sensors toward intelligent artificial skins and propose potential research directions.

## 2. Ionic Materials

Inspired by human skin, mimicking the sophisticated properties of human sensing through electronic approach has become significant for the advancement of artificial intelligence and human interactive electronics [32–35]. To better mimic the mechanical sensing properties of human skin, ionic materials are required to have low Young's modulus for conformability/flexibility, promise high sensitivity to mechanical stimuli, and be capable to convey mechanical sensing information via ionic conduction. Compared with originally utilized liquid-state ionic materials, solid-state soft ionic materials are better candidates for the upcoming intelligent devices by virtue of their excellent stretchability, high transparency, and mechanical conformality. Ionic materials can be categorized into liquid-state, solid-state, and natural ionic materials. In this section, we review the commonly used ionic materials with their specific advantages for high-performance iontronic sensors.

### 2.1. Liquid-State Ionic Materials

Over the past decades, liquid-state ionic materials have become a popular material choice for flexible sensing platforms due to their remarkable interfacial capacitance. Leveraging conductive ionic liquids as the active sensing materials, various ionic sensing devices have emerged [36–39]. In general, liquid-state flexible sensing devices employ microfluidic-based configuration to confine the liquid ionic materials in the flexible elastomeric substrates or templates. Benefiting from their unique advantages (e.g., wide availability, low-power consumption, high sensitivity, and low cost), liquid ionic material-based mechanical sensors have made rapid progress in abundant novel applications.

#### 2.1.1. Aqueous Electrolytes

Aqueous electrolytes are formed through dissolving ionic compounds in water, which can be acidic/alkaline/neutral depending on the concentration of H^+^ and OH^−^ ions. Generally, aqueous electrolytes enable higher power capability compared to organic solvent-based electrolytes due to that the aqueous electrolytes (with higher ionic mobility) exhibit better ionic conductivity by two orders of magnitude. However, the use of aqueous electrolytes is restricted by their narrow electrochemical stability window, which sets an intrinsic limit on the practical voltage and energy output. Besides, aqueous electrolytes are prone to lose their solvent molecules as the result of inevitable evaporation, which substantially decreases the stability of the mechanical sensors. Although aqueous electrolytes have already been widely adopted in supercapacitive and energy storage devices, they are not the best option for long-term mechanical sensors with stability requirement.

#### 2.1.2. Nonaqueous Electrolytes

Considering the instability of aqueous electrolytes, nonaqueous electrolytes comprising organic electrolytes and ionic liquid appear as an alternative. Nonaqueous electrolytes exhibit interesting properties, including low viscosity, nonflammability, nonvolatility, high thermal/chemical stability, and wide operating temperature range [40–42].

Organic electrolytes are typically obtained via dissolving ionic compounds in organic solvents. Nie et al. utilized glycerol/water solution of NaCl as organic electrolyte to form the transparent sensing droplet, employing the electric double layer (EDL) formed at the extremely soft droplet-electrode interface with ultrahigh capacitance as the sensing parameter [31]. The novel droplet sensor exhibits extremely high unit area capacitance (UAC) ranging from 4.4 to 4.7 *μ*F cm^−2^ depending on the mixing ratio of electrolyte/glycerol. The large UAC of EDL at the elastic droplet-electrode interface enables notable sensitivity (1.58 mF kPa^−1^) with ultrahigh resolution (1.8 Pa) based on a simply suspended membrane structure. It is worth noting that surface hydrophobic treatment can be introduced to reduce the droplet-deformation hysteresis without sacrificing the interfacial capacitance substantially. Besides, liquid evaporation problem can be addressed by adjusting the mixing ratio of electrolyte and glycerol, ensuring long-term stability of the mechanical sensor. Even though certain organic solvents possess low saturated vapor pressure to alleviate liquid evaporation, there are still environment-related concerns (e.g., moisture absorption and resultant viscosity problem), which may result in unexpected interfacial properties for iontronic mechanical sensing in varying environments.

Ionic liquids have become a popular choice in iontronic sensors due to their unique properties [40, 43, 44]. Ionic liquids are molten salts comprising of organic cations and organic/inorganic anions (melting points well below 100°C). Recently, ionic liquids have been widely adopted as materials for microfluidic mechanical sensing owing to their great ionic conductivity, excellent thermal/chemical stability, wide operating temperature range, and tunable fluidic viscosity [45–47]. For common ionic liquids, organic salts (e.g., tetraalkylammonium (TAA^+^), imidazolium, pyrrolidinium, piperidinium, and pyridinium, as well as their derivatives) are adopted as cations, while inorganic salts (e.g., tetrafluoroborate (BF_4_^−^), trifluoroacetic (CF_3_CO_2_^−^), dicyanamide (DCA^−^), hexafluorophosphate (PF_6_^−^), and bis(trifluoromethanesulfonyl) amide (TFSI^−^)) are used as anions. Because of the highly controllable molecular design and broad choice in combination of ions, numerous innovative applications have been emerging.

The first experiment to introduce ionic liquids for strain sensors was proposed by Yun et al., in which the maximum true strain can reach 55% [47]. Based on polydimethylsiloxane (PDMS) with microchannel-established gauge structures, they employed high-conductivity ionic liquid ([BMIM][BF_4_]) as the piezoresistive material and carbon fibers as the electrodes. Yoon et al. designed a microfluidic strain sensor with highly stretchability and transparency taking advantage of the merits of both ionic liquids and microfluidic system [45]. The binary mixture of ionic liquid ([BMIM][Ntf_2_] and [BMIM][Ac] with the ionic conductivity of 3.9 mS·cm^−1^ and 1.9 mS·cm^−1^, respectively) is filled to the microfluidic channels embedded in elastomer PDMS slabs. The maximum strain of the developed microfluidic strain sensor reaches up to 200% with a gauge factor of 2 in the linear region (strain < 50%) and a gauge factor of 40 under maximum stretching (200% strain). Additionally, the microfluidic strain sensor successfully realizes real-time human motion monitoring. Based on piezoresistive mechanism, there are considerable reports for iontronic strain or pressure sensor selecting ionic liquids as piezoresistive materials because of their good mechanical deformability and electrical reversibility. However, there are still some challenges in the further development of the microfluidic resistive strain sensors for ionic liquids: (i) hysteresis problem resulting from the viscoelastic characteristic of elastomeric microfluidic channels, (ii) the contradiction between stretchability and sensitivity, and (iii) temperature interference in microfluidic resistive strain sensors.

To overcome these problems, supercapacitive sensors based on ionic liquids have gained lots of attentions because of their ultrahigh sensitivity, low-power consumption, facile structure, and wide operating temperature range. A supercapacitive iontronic microdroplet array was first reported by Nie et al. to achieve flexible tactile sensing, by leveraging the ultrahigh interfacial capacitance at the soft droplet-electrode contact [48]. Therein, ionic liquid nanoliter droplet sensors are sandwiched between two elastic polymer membranes with transparent conductive electrodes. As the droplet sensing material, [EMIM][TCM]-based ionic liquid has shown high electrical conductivity (18 mS·cm^−1^) and low viscosity (18 Pa·s). Benefiting from ultrahigh UAC of the iontronic microdroplet, the sensory device has achieved excellent sensitivity of 0.43 nF·kPa^−1^ with resolution at 33 Pa. The iontronic microdroplet array device is also successfully attached onto the fingertip to map the delicate topologies of fingertip skin. Besides, dynamic pressure curves throughout cardiovascular cycles can be obtained embedding the device into a wristband. To further exploit the iontronic mechanical sensors for multidirectional contact force detection, a microfluidic tactile sensor is designed to measure both the normal mechanical forces (along the *z-*axis) and the forces tangent to the surface (*x*‐*y* axes) when in contact [49]. The working mechanism can be described as follows: the microtextured membrane surface subjected to the normal (or shear) forces leads to the deformation of underneath ionic liquid uniformly (or differentially). These two processes result in distinguishable interfacial capacitance variations to indicate the magnitude and direction of the external force. According to the ultrahigh interfacial capacitive sensing mechanism, the microfluidic tactile sensor can achieve excellent pressure sensitivity of 29.8 nF·N^−1^ for normal force detection and 2.12 nF·N^−1^ for shear force detection, respectively.

Overall, ionic liquids are one of the most promising candidates for liquid-based iontronic sensors with high sensitivity, low volatility, and excellent environmental stability.

### 2.2. Solid-State Ionic Materials

**Figure 2:**
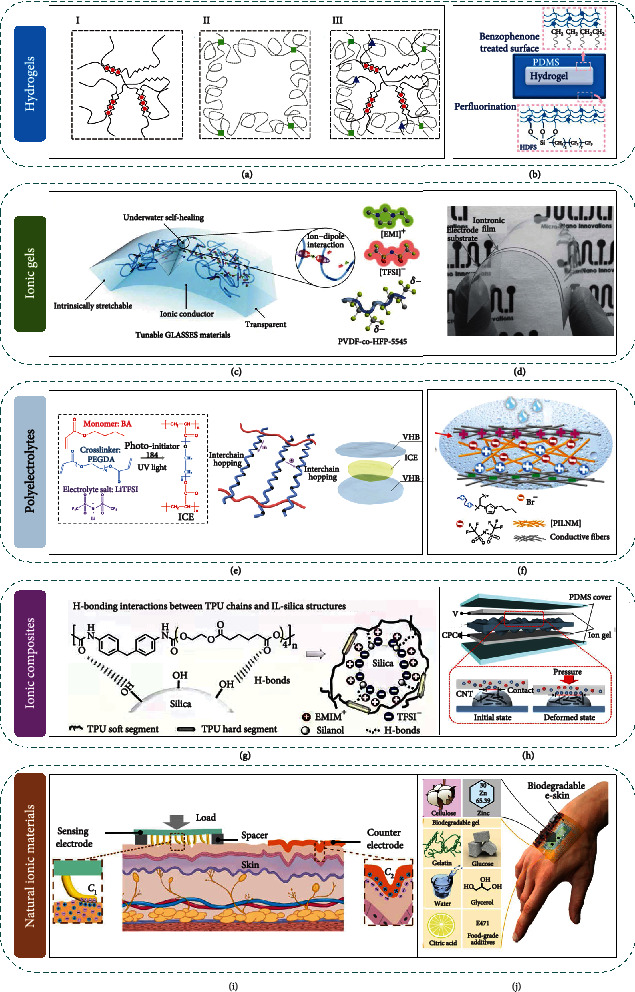
The category of the materials used in iontronic sensors. (a) Schematics of intertwined hybrid hydrogel networks (III), ionically crosslinked alginate networks (I), and covalently crosslinked polyacrylamide networks (II) [64]. Reproduced with permission, copyright 2012, Nature Publishing Group, a division of Macmillan Publishers Limited. (b) Cross-sectional structure of fabricated self-cleanable, transparent, and attachable ionic communicators [65]. Reproduced with permission, copyright 2018, The Author(s). (c) Design of a gel-like, aquatic, stretchable and self-healing electronic skin (GLASSES) [78]. Reproduced with permission, copyright 2019, The Author(s), under exclusive license to Springer Nature Limited. (d) The interfacial capacitance change caused by the ionic gel mixture ratio, from 25 to 67 wt% of the ionic liquid component, given a constant film thickness of 110 *μ*m [80]. Reproduced with permission, copyright 2015, WILEY-VCH Verlag GmbH & Co. KGaA, Weinheim. (e) Molecular structures of precursors and the polymerized ion-conducting elastomer [85]. Reproduced with permission, copyright 2020, WILEY-VCH Verlag GmbH & Co. KGaA, Weinheim. (f) Schematic illustration of a self-assembled PILNM-based wearable and washable poly(ionic) nanofibrous membrane with moisture proof pressure sensing [86]. Reproduced with permission, copyright 2019, American Chemical Society. (g) A bioinspired hydrogen bond-triggered ultrasensitive ionic mechanoreceptor skin [90]. Reproduced with permission, copyright 2019, The Author(s). (h) Schematic of ionic carbon nanotube/poly(dimethylsiloxane) composite pressure sensor [91]. Reproduced with permission, copyright 2017, American Chemical Society. (i) Exploded view and schematic illustration of the skin-electrode sensing structure [95]. Reproduced with permission, copyright 2021, The Author(s). (j) Naturally derived ingredients, such as gelatin and citric acid, enable an elastic and stable, but fully degradable, biogel, and multifunctional ionic skins are realized [104]. Reproduced with permission, copyright 2020, The Author(s), under exclusive license to Springer Nature Limited.

As aforementioned, liquid-state ionic materials have shown great potential in iontronic sensors by the merits of excellent stretchability, high transparency, and mechanical conformality. However, the liquid nature meanwhile restricts its further applications due to poor stability, nonportability, and difficulty in miniaturization. In this regard, ideal iontronic sensors are required to possess the primary high conductivity and high sensitivity with additional mechanical conformality. Solid-state ionic materials have arrested considerable attentions in terms of their high stretchability, mechanical conformality, and excellent conductivity/transparency, which compensate the drawbacks of liquid ionic materials. They are classified into typical hydrogels, ionic gels, polyelectrolytes, and ionic composites in the following discussions (Figure 2, specific natural ionic materials will be discussed in Section 2.3).

#### 2.2.1. Hydrogels

Hydrogels are formed through hydrophilic polymer networks crosslinked and swollen with water. The polymer network forms the elastic solid framework of hydrogel, while the water molecules promise hydrogel to be an ionic conductor [50–55]. Conductivity of hydrogel can be improved from ~5.5 × 10^−8^ S/m (the value of purified water) to ~10 S/m by dissolving different types/concentrations of salts in water to yield mobile ions [56]. Since the pioneering report [57] by applying hydrophilic gels for biological use, hydrogels have been widely exploited as brand new ionic conductors in soft electronics [58, 59] by virtue of skin-like tactile impression [60], excellent stretchability, super transparency, outstanding conductivity, and high sensitivity [61–63].

One of the most prominent examples is the extremely tough and stretchable hydrogel synthesized by Sun et al. through combining covalently crosslinked polyacrylamide and ionically crosslinked alginate [64]. Figure 2(a) shows the schematic illustration of three typical polymer networks of hydrogel, including ionically (I), covalently crosslinked (II), and intertwined double networks (III). When the hydrogel is stretched, the ionically crosslinked alginate can be fractured to eliminate the strain energy substantially, while the covalently crosslinked polyacrylamide enables the hydrogel to recover to the initial state. The intertwined double networks endow the hydrogel with high resistivity to defects, achieving a stretchability as high as 1700% even with a notch.

Owing to the excellent mechanical stretchability and stable physicochemical properties, hydrogels have been utilized to build various human-machine interaction platforms with the delicately designed iontronic sensors. A hydrogel-based self-cleanable ionic communicator is fabricated by Lee et al. to exploit an attachable human-machine interface [65] (Figure 2(b)). The chemical bonding at the interface between the AAm-LiCl hydrogel and PDMS endows the ionic communicator with mechanical reliability. In addition, surface treatment by (heptadecafluoro-1,1,2,2-tetrahydrodecyl) trichlorosilane (HDFS) ensures the contact interface to be clean without sacrificing transmittance and durability. The ionic communicator is characterized with high transparency (99.6%) and stretchability (330%). Further applications for touch detection are explored for the self-cleanable and attachable ionic communicators to input alphabets in computer assisted with wireless emitters. Based on capacitive touch sensing, PAAm–NaCl hydrogel has also been fabricated into cross-grid array as an ionic touch panel to capture finger proximity and multiple touches [66]. Besides, hydrogels are also the best-known transparent soft conductors. The excellent mechanical and physicochemical characteristics promise the hydrogel to be the next-generation iontronic sensors.

Except for mechanical stretchability, the integration of other novel functions into materials (e.g., self-healing ability) is urgently required as the ionic skin inevitably suffers from risky damages, which may cause failure of the device. Such properties commonly rely on the capability to reestablish molecular interactions after the materials are subjected to external forces or breakage. Inspired by human muscles, Ge et al. designed a hydrogel-based self-healing strain sensor [67]. The hydrogel-based sensor exhibits high gauge factor of 18.28 in a broad strain range (268.9% strain) and low detection limit (5% strain). Resulting from the excellent mechanoreceptive and thermosensitive properties, a self-healing touch keyboard for signature recognition is achieved by the hydrogel bioelectronic device. Xue et al. proposed a single-layer hydrogel ionic skin with ultrahigh stretchability (7700%), self-healing properties, and ultrahigh sensitivity to mechanical stimuli (gauge factor of 1.39) [68]. The device also allows self-healing of both mechanical and electrical properties after damage. These novel characteristics are expected to significantly improve the performance of hydrogel-based ionic skin and promote the construction of complex hydrogel-based wearable devices.

Based on the discussions in this section, it is worth noting that hydrogels are the most promising candidates for multifunctional iontronic sensors. Hydrogels can not only maintain liquid-like properties but also exhibit mechanical conformality, which is essential for long-term stability of the devices. Specially, ultrahigh stretchability and self-healing capabilities are highly desirable for the soft artificial skins and human interactive interfaces. However, under dry conditions, water evaporation of the hydrogels compromises the stability of hydrogel-based devices, which may limit their applications [69]. Adding hygroscopic matter and elastomeric coating materials on the hydrogels can help alleviate the evaporation of the hydrogel.

#### 2.2.2. Ionic Gels

Considering the water evaporation problem of the hydrogel, ionic gels have emerged as an alternative option for ionic skin and artificial intelligence [70–77]. Ionic gels are typically formed by swelling polymer networks in ionic liquids. As the polymer molecular chains are connected or entangled with each other, a spatial polymer network structure with the interspaces filled with anions and cations is formed (can be considered as a dispersion medium). Merging the high ionic conductivity of ionic liquids with the polymeric matrix with the mechanical conformality, the ionic gels have been extensively employed in iontronic sensors.

Similar to hydrogels, ionic skins composed of ionic gels have made great achievements accounting for the similar touch impression to human skins. Inspired by transparent jellyfish, Cao et al. devised a submersible self-healing ionic skin [78] (termed as “GLASSES”), which is composed of an amorphous polymer (PVDF-co-HFP-5545) and a chemically compatible ionic liquid ([EMIM][TCM]) (Figure 2(c)). The stretchable, gel-like, aquatic, and self-healing ionic skin owes ionic conductivity up to 10^−3^ S·cm^−1^ and can realize autonomous self-healing in both dry and wet environments owing to reversible ion-dipole interactions (by mimicking the self-healing behavior of human skins). Even in acidic or alkali environments, the GLASSES can realize rapid and reproducible electromechanical self-healing with the healing efficiency of 99.99%. The excellent self-healing performance under various environments is due to the modification by fluorinated polymers, which are known to be stable under different conditions, such as salty and different pH. In addition, the hydrophobicity of the ionic liquid synergistically shields it against the water molecules. The GLASSES (stretchability as high as 2000% and transmittance of 98%) is demonstrated as capacitive touch, pressure, and strain sensors for further human interactive applications. To improve the sensing performance, Qiu et al. introduced surface microstructures into the ionic gel-based artificial skin [79]. The microstructured ionic gel film is replicated from Calathea zebrine leaf via soft lithography techniques, thus creating uniform cone-like microstructures on its surface. The microstructured ionic skin shows a detection limit as low as 0.1 Pa. Significantly, a superior sensitivity of 54.31 kPa^−1^ in the low-pressure range (<0.5 kPa) has been achieved. With pressure in a broader range (from 0.1 to 115 kPa), the sensitivity is still larger than 1 kPa^−1^, which is beneficial for subtle and fine perceiving of the ionic skin.

Regarding emerging wearable and healthcare sensing applications, Nie et al. fabricated the first ion gel-based pressure sensor for real-time pulse pressure waveform detection [80] (Figure 2(d)). The stretchable and transparent ion gel is prepared by the UV-crosslinkable poly(ethylene glycol) diacrylate (PEGDA) polymer networks and the ionic liquid of [EMIM][TCM]. Accounting for the established EDL, the sensor possesses an ultrahigh specific capacitance of 5.4 *μ*F·cm^−2^, which results in an ultrahigh sensitivity of 3.1 nF·kPa^−1^ (three orders of magnitude higher than that of conventional solid-state counterparts) according to the mechanical to capacitance mechanism. In addition, ultrafast response in submillisecond range is successfully achieved. The sensor can be attached to the finger to track blood pressure continuously and even resolve fine surface topology, which is very important for further development of wearable healthcare sensing. Another work reported by Li et al. demonstrates a telemedical wearable sensing platform based on ionic gel, which promises for the long-term home care and remote clinics [81]. The ionic gel is composed of PEG polymer networks and ionic liquid [EMIM][TCM]. For interface pressure measurement, the iontronic sensor is characterized with a sensitivity of ~0.2 nF/mmHg based on the principle of interfacial capacitive sensing. The wearable sensing platform provides the users to achieve both clinical and individual judgment and management of chronic venous disorder.

The material characteristics of the ionic gel are similar to those of biological tissues but exhibit conductivity (like metals or semiconductors). It promises the ionic gel to make a big splash in the fields of biosensors and intelligence biomedical devices.

#### 2.2.3. Polyelectrolytes

Polyelectrolytes are an important category in solid electrolytes, which contain potentially ionizable groups on their structural units [82–84]. Normally, the ionic conductivity of polyelectrolytes is completely dependent on the mobility of the ionized species. Zhang et al. reported an iontronic tactile sensor based on triboelectric nanogenerator (TENG) by incorporating a photopolymerization process [85]. In the polyelectrolytes (Figure 2(e)), BA monomer acts as photoinitiator and PEGDA serves as the crosslinker to form the solid-state polymer. LiTFSI provides the highly conductive component which ensures high capacitance and remarkable sensitivity for iontronic mechanosensing. The ion transfer is resultant from the intrachain or interchain hooping process (i.e., breaking/forming lithium–oxygen bonds) within the ion-conducting elastomer chains. The polyelectrolyte iontronic sensor, with high stretchability (1036%), transparency (91.5%), and thermally stability (storing at 100°C for 15 h), exhibits an excellent sensitivity up to 2.87 kPa^−1^ and a low detection limit of 400 Pa. The ionic TENG shows dual functions of biomechanical energy harvesting and pressure sensing, suggesting the potential application as self-powered and highly sensitive pressure sensor. The demonstrated ionic TENG is favorable for future smart, energy-efficient, and low-cost ionic skins and wearable electronics.

By integrating iontronic devices into textiles, Wang et al. first fabricated a flexible and washable iontronic sensing textile for wearable intelligent electronics [86]. Through the free radical polymerization of [PBVIm][TFSI] and subsequent electrospinning process (Figure 2(f)), nanofibrous polyelectrolyte membrane is prepared with outstanding mechanical properties. The ionic textiles with microporous structures are ideal polymer dielectrics to amplify the subtle and fine stimuli signals, leading to high sensitivity at 0.49 kPa^−1^ and low detection limit at 20 Pa. This is because the microporous assemblies in the ionic textiles can help to squeeze out the air and elevate the volume fraction. Thus, a small initial volume fraction or small initial contact area of micro-/nanostructured iontronic layer allows a larger area fraction change under compression to amplify the subtle and fine stimuli signals. Notably, [PBVIm][TFSI] is also hydrophobic with good resistance to moisture, which enables the textiles to be washable. According to the superior performance of the iontronic sensor, the assembled wearable ionic textile can be applied to monitor various human motions and subtle pulse vibrations.

Polyelectrolytes are promising materials for iontronic sensors, but their conductivity is relatively low. Even selecting high-conductivity electrolyte salt, the polyelectrolytes still exhibit a low conductivity, three orders of magnitude lower than ionic gels and hydrogels. Besides, the sensitivity to environmental humidity is another problem that needs to be overcome.

#### 2.2.4. Ionic Composites

Combining multiple material components into iontronic sensors can provide new ideas for further research on the revolution of multifunctional sensing applications [87–89], which either compensates for the shortcomings of the original materials or introduces additional functions.

Inspired by biological cellular structures, Amoli et al. constructed a hydrogen bond-triggered ionic mechanoreceptor by introducing silica microstructures [90] (Figure 2(g)). After confining the ionic liquid [EMIM][TFSI] on silica microstructures, an artificial plasma membrane geometry is achieved and embedded into TPU polymer matrix to mimic the multicellular structures. The working mechanism is different from conventional ionic sensor. Initially, most ions of [EMIM][TFSI] are confined on silica microstructures resulting from the H-bond–co-Coulomb interactions. When the ionic mechanoreceptor is subjected to external stimuli, reversible ion pumping effect results in the formation of EDL at the [EMIM][TFSI]–silica–TPU/electrode interface to implement the sensation process. The bioinspired ionic skin is ultrasensitive (48.1–5.77 kPa^−1^) over a wide pressure range (from 0 to 135 kPa), which is superior to various pressure sensors and natural skin mechanoreceptors. By embedding CNTs into ionic gels, Yoon et al. have devised an alternative iontronic pressure sensor capable of detecting both the vertical pressure and horizontal movement in applied force [91] (Figure 2(h)). The pressure sensor is prepared by filling ([EMIM][TFSI]) ion liquids in P(VDF–HFP) film and microbump-structured CNT/PDMS nanocomposite film (serving as electrode and dielectric layer, respectively). After evaluating the sensing performance based on symmetric and asymmetric configurations, a reliable pressure sensitivity gradient is observed in the asymmetric configuration due to different capacitance changes, which enables the lateral pressures to be monitored. The resulting capacitive pressure sensors yield a relatively high sensitivity of 9.55 kPa^−1^. Introduction of the irregular microbump-structured CNT/PDMS into iontronic pressure sensor not only increases the conductivity of the polymer but also improves the sensitivity.

Besides leveraging extraordinary properties of the material, unique structure of the material also contributes to improve the performance of the ionic composite-based sensors. Utilizing the unique structural property of polyurethane sponge, an iontronic capacitive pressure sensor is developed by Yang et al. [92] The iontronic sensor is prepared by filling the ionic liquid ([EMIM][TFSI]) into the polyurethane sponge, in which the porous and elastic properties provide greater possibility in the variation of contact area and distance between two electrodes. The innovative iontronic sensor shows a high sensitivity of 5.28 nF·kPa^−1^ and a fast dynamic response (100 ms).

On the other hand, introducing functional particles can bring additional functions, which is essential for developing multimodal iontronic sensing applications. Based on the mechanoluminescent property of the added ZnS, Liang et al. designed a dual-modal ionic skin which can realize touch-sensing and exteroception-visualizing [93]. The stretching state can be indicated by both electric output signal and the light-emitting intensity. Under external stimuli, the electrical output increases with the decreased light-emitting intensity because the mechanoluminescent intensity is positively correlated to the distribution density of ZnS particles. Relevant strategies of introducing functional material particles can promote the iontronic sensors toward multimodal smart artificial skins.

### 2.3. Natural Ionic Materials

Remarkably, ionic materials are ubiquitous in natural world, which are whether liquid-state ionic materials or solid-state ionic materials. Naturally existing iontronic sensing mechanism offers an efficient way to developing sophisticated iontronic sensors. Natural ionic materials are also superior candidates for iontronic sensors, which can be readily achieved without the need for synthesis. The natural ionic materials show superior biocompatibility compared with artificial ionic materials, allowing for eco-friendly processing and applications (some are even edible).

Human skin is one of the most common natural iontronic mechanical interfaces. According to its working mechanism, one of the pioneering epidermal iontronic interfaces is proposed by Zhu et al. It adopts single-sided device and the human skin to constitute the pressure sensing architectures, enabling transparent, ultrathin, and imperceptible properties with fully conformal epidermal contact [94]. It is worth noting that the iontronic interface contributes to ultrahigh sensitivity (5 nF·kPa^−1^), high signal-to-noise ratio, and ultralow motion artifacts for detecting both body physiological signals and environmental mechanical stimuli.

Another interesting work incorporates the skin of living system to construct an iontronic sensing architecture, as depicted in Figure 2(i). Zhu et al. proposed the skin-electrode iontronic interface mechanism for sensing [95]. Given that the skin owes internal ionic transport capability in living systems which serves as a natural ionic interface, the skin-based iontronic sensors greatly simplify the sensing mode and alleviate the use of artificial ionic materials. The skin-electrode mechanosensor simply adopts the skin and a microstructured sensing electrode, which enables subtle signal detecting and conformal measurements. A high-sensitivity (1.3 kPa^−1^ with pressure under 3 kPa, 11.8 kPa^−1^ with pressure within 3-4 kPa, and 2.8 kPa^−1^ within 4-15 kPa) and high-spatial-resolution (submillimeter) tactile sensing function can be realized by simply attaching electrodes to the skin surface. The above sensing mode has great potential to be extended to other living systems, offering a brand-new idea for epidermal sensing technology and wearable intelligent electronic technology.

Apart from human skin, other natural ionic materials (e.g., hyaluronic acid, alginate, protein, chestnut, wood, and cellulose) [64, 96–103] have become hotspots for emerging iontronic sensors due to their remarkable availability (Figure 2(j)). A versatile gelatin-based natural material is developed by Baumgartner et al., which is highly resilient but can fully degrade when disposed [104]. Specially, cellulose (a prevalent structural polysaccharide) is explored to serve as an exoskeleton for soft pneumatic iontronic actuators, which allows dynamic detection. Incorporating the biogel with structured zinc electrodes enables fully degradable iontronic sensor, paving an eco-friendly way for future sensory devices which mimic human skin.

The summary of materials, working mechanisms, and applications of recently reported iontronic sensors is summarized in details as displayed in Table 1.

## 3. Working Mechanisms of Iontronic Sensors

Effective signal transductions which convert external stimuli into analog electronic signals are critical for accurately quantitative monitoring. Conventional transduction mechanisms (e.g., capacitive, resistive, and piezoelectric) are widely explored in various mechanical sensors, while other emerging transduction mechanisms (e.g., electroluminescent, electrochromic, and triboelectricity) are undergoing dramatic revolution to satisfy upcoming opportunities and challenges that will tremendously extend the potential applications of ionic skins toward prosthesis, robotics, human interactive interface, and artificial intelligence. In this section, the details of various working mechanisms for iontronic sensors are provided.

### 3.1. Interfacial Capacitive Sensing

**Figure 3:**
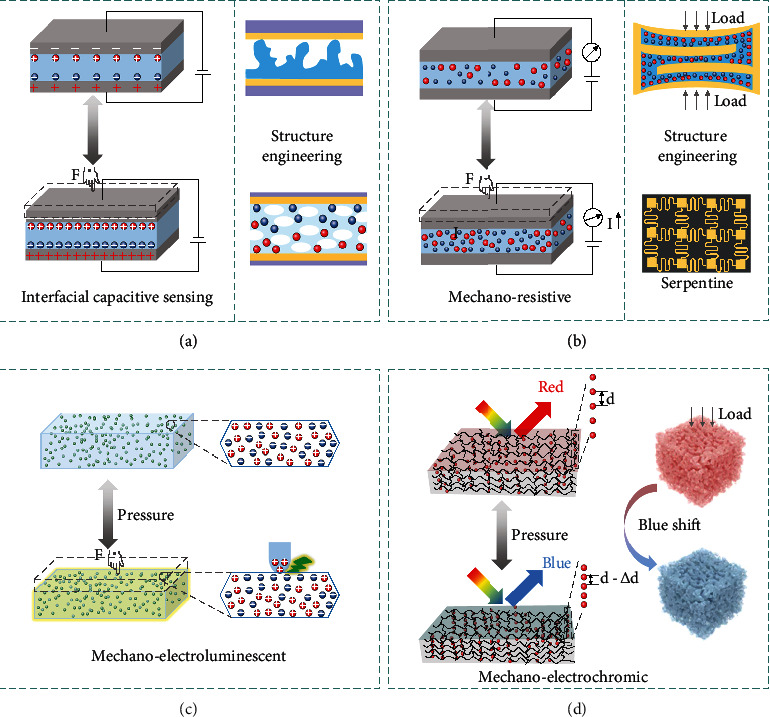
Working mechanisms of ionic sensing devices. (a) Interfacial capacitive sensing. (b) Mechanoresistive. (c) Mechano-electroluminescent. (d) Mechano-electrochromic.

Interfacial capacitive sensing lies in the conversion of external force vibrations into the variation of EDL capacitance. Through establishing EDL at the ionic dielectric/electrode contact (theoretically with an ultrathin thickness of ~1 nm), the capacitance of iontronic devices can be notably promoted, which remarkably elevates the interfacial capacitive effect under pressure (an ultrahigh UAC up to several *μ*F·cm^−2^). Typically, once the ionic material with considerable mobile cations and anions (liquid or solid) contacts with the electrode, the charged electrode will repel the coions and attract the counterions from the inside of ionic material to the interface. Thus, two EDLs are established at the electronic/ionic interfaces (Figure 3(a)).

In principle of the classic EDL model, the immediate interfacial layer in the ionic material confining counterions in nanometer ranges is defined as Helmholtz layer, while the adjacent layer is known as diffuse layer. The Helmholtz layer and diffuse layer owe their individual capacitive elements, which is *C*_*H*_ and *C*_*D*_, respectively. The equivalent model of the interfacial EDLs can be regarded as two interfacial capacitors in series. The EDL capacitance (*C*_EDL_) can be theoretically expressed by [115]

(1) $$\begin{array}{c} C_{\text{EDL}}={\left(\frac{1}{C_{H}}+\frac{1}{C_{D}}\right)}^{-1}. \end{array}$$

Given that both *C*_*H*_ and *C*_*D*_ are proportional to the contact area between the electrodes and ionic interfaces, *C*_EDL_ is straightforwardly bound up with the contact area and correlation coefficient (UAC). Specifically, the value of unit area capacitance is influenced by various factors (e.g., ionic species, ionic concentrations, surface potential, and temperature). In ideal conditions, that is, considering the unit area capacitance as a constant, the sensing mechanism of interfacial capacitance is experimentally determined by the mechanically induced changes in interfacial contact area, which brings about the capacitive change in iontronic sensors.

The first EDL interfacial capacitance model is proposed by packaging the ionic droplet with a polymeric separation layer. The capacitive sensitivity of the sensor can be analytically derived into [31]

(2) $$\begin{array}{c} \frac{\Delta C_{\text{EDL}}}{P}=C_{0}\left(\alpha R^{2}+\beta H\right)\frac{V_{d}R^{2}}{H^{2}}, \end{array}$$

where *C*_0_ is the unit area capacitance and *α* and *β* stand for the membrane deflection and the elastic deformation of the separation layer, which are dependent on the geometrical and mechanical performance of sensing membrane and separation layer, respectively. *R* and *H* indicate the radius and height of sensing chamber, and *V*_*d*_ represents the volume of the ionic droplet.

Another work demonstrates the pressure-capacitance relationship of gel-based mechanosensor. Upon compression, the PDMS/TLC dielectric of the ionic sensor will be thinned down, resulting in an increased capacitance accordingly, which can be determined by [116]

(3) $$\begin{array}{c} C_{\text{EDL}}=\varepsilon_{r}\varepsilon_{0}\frac{\pi r^{2}}{d}=\varepsilon_{r}\varepsilon_{0}\frac{\pi r_{0}^{2}}{d_{0}}\frac{E^{2}}{{\left(E-P\right)}^{2}}, \end{array}$$

where *ε*_*r*_ is the relative permittivity of the PDMS/TLC composite, *ε*_0_ is the permittivity of free space, *r*_0_ is the radius of the unpressed, *d*_0_ is the thickness of the device, *E* is the elastic modulus of the gel, and *P* is the applied pressure.

Regarding to the nanostructured ionic capacitive sensing, a capacitive pressure sensing model has been derived from the classic compression theory of fibrous assemblies. Being subjected to compression, the fibrous assemblies squeeze out the air and elevate the volume fraction, leading to an increment in contact area between the electrodes and the ionic region. The compression induces the variation of fiber area fraction, which will lead to the interface capacitance change in EDL. The relationship can be expressed as [117]

(4) $$\begin{array}{c} C_{\text{EDL}}=C_{0}\frac{A}{2\pi }\left[{\left(\frac{P}{KE}+V_{f_{0}}^{3}\right)}^{1/3}-V_{f_{0}}\right], \end{array}$$

where *C*_0_ is the unit area capacitance, *A* is the contact area, and *K* is related to the spatial distribution and properties of the fiber (constant for a given material). *E* is the fiber's elastic modulus, and *V*_*f*0_ is the volume fraction of the fiber assembly at the initial state. According to Equation (4), a small initial volume fraction or small initial contact area of nanostructured iontronic layer allows a greater area fraction change under compression, which is the key to obtain high pressure sensitivity in iontronic sensors. Besides, ionic materials with lower Young's modulus are favorable to gain high sensitivity.

Notably, although ionic materials with lower Young's modulus offer excellent flexibility and compressibility for iontronic capacitive sensing, the deformation of ionic materials will inevitably reach a saturation state during pressure sensing process and result in a limited sensitivity. Considering the material limitation, structural engineering is an effective approach to further improving the sensing performance of iontronic capacitive sensing. There are various structure engineering methods, e.g., replicating surface microstructures, introducing internal microporous structure, and designing interlock structures.

Surface microstructure engineering (e.g., micropyramids, microdomes, micropillars, and hierarchal microstructures) normally leverages 3D surface microstructure design of the ionic polymer matrix to improve sensitivity, selectivity, sensing range, response time, mechanical compliance, and device pixel resolution. Through creating gaps between the ionic polymer matrix and electrodes, surface microstructure engineering not only enables a small initial contact area but also facilitates the large variation of contact area. Based on surface structure engineering, Bai et al. exploited sandpaper as a mold to produce an ionic gel membrane with high aspect ratios and fillable structures [118]. The designed structure presents a fractal surface, which can be easily deformed and then filled in the groove when subjected to an external mechanical force. Benefitting from the delicately designed groove, more space is achieved to allow further compression by accommodating deformed microstructures. The intrafillable iontronic sensor exhibits an unprecedentedly high sensitivity of *S*_min_ > 220 kPa^−1^ over a broad pressure regime (0.08 Pa-360 kPa), an ultrahigh pressure resolution (18 Pa or 0.0056%) over the full pressure range, and remarkable mechanical stability.

Unlike surface structure engineering, the internal microporous structure engineering utilizes microporous structures inside the ionic polymer matrix to improve its Young's modulus. When subjected to the same external mechanical force, the iontronic capacitive sensor with internal microporous structures can lead to a greater capacitance change. The effective dielectric constant increases with the gradual shrinkage of the micropores. During the unloading process, the intrinsic stress of the elastic ionic material reopens the microporous structure, which allows the capacitance of the iontronic sensor to recover to its initial state. Moreover, microporous structures can provide sufficient space to suppress the bucket phenomenon (a phenomenon appearing in classical capacitive interfacial iontronic sensors). Interlocked structure is another interesting structure engineering strategy [119]. By introducing interlinked and microstructured interfaces in a multilayered sensor, the strong topological interlinks between different functional layers can lead to tough interfaces with larger contact area change upon applied pressures, which are promising to improve the sensitivity.

The ultrahigh sensitivity of the EDL-based iontronic mechanosensor promises the interfacial capacitive sensing to have a hunting ground in high-precision ionic skins, human-machine interactive interface, and artificial intelligence.

### 3.2. Mechanoresistive

Mechanoresistive sensors enable conversion of external force variations into resistance changes. The working mechanism is explained as follows. When electric field is applied to the mechanoresistive sensors, cations and anions in ionic materials will migrate toward cathode and anode, forming ionic currents in the ionic materials. The external stimuli can lead to change in cross-sectional area and length of the ion-conducting channels, resulting in a change of resistance. The most common methods to acquiring the pressure dependence resistance change include contact resistance change (between conductive materials and electrodes) and conductive path change (in conductive elastic composites). As depicted in Figure 3(b), the external stimulus allows an increased cross-sectional area of the ion-conducting channels and decreased conducting channel length, resulting in an increased ionic current according to the classic resistive model. The resistance of the iontronic sensor can be described in the following [120]:

(5) $$\begin{array}{c} R^{\prime }=\rho \frac{L\left(1+\varepsilon \right)}{A^{\prime }}, \end{array}$$

where *ρ* represents the electrical resistivity of ionic materials, *L* is the length, *ε* is the strain, and *A*′ is the cross-sectional area. In particular, for liquid ionic sensor with microchannels, the theoretical relationship between resistance variation (Δ*R*) and strain (*ε*) can be expressed by [121]

(6) $$\begin{array}{c} ∆R=\frac{\rho L}{wh}\left\{\frac{\left(1+2\mu \right)\varepsilon -\mu^{2}\varepsilon^{2}}{{\left(1-\mu \varepsilon \right)}^{2}}\right\}, \end{array}$$

where *ρ* is the resistivity of ionic materials, *w* and *h* are the width and height of the cross-section of the microchannel, *μ* is the Poisson's ratio of the elastomer material, and *ε* is the strain.

To evaluate the strain sensitivity, a gauge factor (GF) is defined in [122]

(7) $$\begin{array}{c} \text{GF}=\frac{R_{S}-R_{0}}{R_{0}\varepsilon }, \end{array}$$

where *R*_0_ and *R*_*S*_ represent the resistance under initial and stretched state and *ε* is the strain.

Similar to iontronic capacitive sensors, structure engineering strategy is also compatible with iontronic resistive sensors. Except for surface structures, internal structures, and interlocks, microchannel and serpentine designs are alternative strategies to improving the sensing performance of iontronic resistive sensors. Microchannel design in iontronic resistive sensing refers to filling elastomeric microchannels with ionic materials. Because the microchannel dimensions can be easily distorted upon external forces applied to the elastomeric polymer layer, the conductive ionic liquids confined inside the microchannel are readily deformed. Thus, the electrical resistance across the channel will change and promises the microfluidic channels (or networks) to be suitable for strain sensing. Commonly, simple linear channels are more frequently used than grid network channels to minimize the complexity of microfluidic system as effective strain sensor. Serpentine structures can further enhance the sensing performance through improving the stretchability of iontronic resistive sensor [123]. As an additional benefit, serpentine structure enables multidirectional force detection along *x*-, *y*-, and *z*-axis.

Owing to the simple architecture and facile testing instrument, mechanoresistive sensing is frequently explored and employed in a wide range of applications.

### 3.3. Mechano-Electroluminescent/Electrochromic

Biological skin systems are capable to perceive multimodal external stimuli through ion transduction. Interestingly, certain advanced organisms (e.g., cephalopods, chameleon, and flatfish) can further swiftly alter their skin color via manipulating photonic nanostructures. Mechano-electroluminescent and mechano-electrochromic mechanisms can be readily used for multimodal perceiving with color-switching phenomena inspired by nature.

Mechano-electroluminescence is a light emission phenomenon when a solid material is subjected to pressure. Typically, mechano-electroluminescence is based on a unique transduction principle from stress to photons, which is promising in advanced stress sensing and multimodal sensing (Figure 3(c)). Remarkably, the emission intensity shows a linear increment with increased pressure (there is no particles fracture or surface damage). To realize good visualizing capability, mechanoluminescent materials should be evenly dispersed into the ionic materials to achieve efficient stress-transferring, so as to ensure an effective light emission when being subjected to external mechanical stimuli.

Furthermore, by embedding mechano-electroluminescent additives into stretchable ionic materials, the iontronic sensors can be endowed with prompt light emission responses to external mechanical stimuli while maintaining high sensitivity to touch sensing [124]. The mechano-electroluminescent sensing compensates the defects of conventional sensing technologies that the sensing results cannot be visually recognized in real time. Multimodal iontronic sensing offers a novel route to diversifying iontronic sensors in mimicking human skins.

The illuminance of mechano-electroluminescent sensor increases with the increased pressure, accounting for the increment of phosphor particles in volumetric number density when the electrode area increases. The relative illuminance (*I*/*I*_0_) can be simulated by [113]

(8) $$\begin{array}{c} \frac{I}{I_{0}}={\left(\lambda_{1}\lambda_{2}\right)}^{-1}\exp\left[a\left(1-{\lambda_{3}}^{1/2}\right)\right], \end{array}$$

where *α* is the fitting parameter and *λ*_1_, *λ*_2_, and *λ*_3_ represent the length change (*L*/*L*_0_) along the axial, transverse, and out of plane orientations, respectively.

Another interesting photonic mechanism upon mechanical stimuli is mechano-electrochromic effect, which can promptly respond to the mechanical stimuli by switching colors. Typically, the iontronic mechano-electrochromic devices are developed by embedding photonic crystals into ionic matrix, combining the mechano-electrochromic property of photonic crystals and the fascinating ionic conductivity of ionic materials with excellent compatibility. The skin-like chromotropic iontronic device can satisfy the requirement of electrical response and optical visualization to mechanical stimuli synchronously via adjusting the ultrastructure as well as ionic mechanotransduction. As illustrated in Figure 3(d), under certain pressure, the thickness along the viewing direction shrinks perpendicular to the stretching plane. Thus, the reflection wavelength *λ* blue-shifts with decreased *d* in principle of Bragg equation [110]:

(9) $$\begin{array}{c} \lambda =2nd\;\sin\theta , \end{array}$$

where *n* is the effective refractive index, *d* is the lattice spacing, and *θ* indicates the incidence angle.

To describe the effectiveness of the mechano-electrochromic sensor to reflect the optical response to external stimuli, the mechano-electrochromic sensitivity is defined by [125]

(10) $$\begin{array}{c} S=\frac{∆\lambda }{∆\varepsilon }, \end{array}$$

which is expressed by the shift in the stopband wavelength (Δ*λ*) according to the applied strain change (Δ*ε*). Normally, the mechano-electrochromic sensitivity (ranging from 0.7 to 5.3) is highly related to the designed materials (especially the mechanical properties of the materials). Besides, the photonic crystal-based interconnected microporous network structure provides an extraordinary environment for ion transport, which guarantees the stability of mechanical-electrical detection.

Accordingly, embedding the mechano-electroluminescent/electrochromic additives into the ionic materials can be an effective approach to endowing the iontronic sensors with the capability of quantitatively feeding back to external stimuli through both electrical and photonic signals (in-visual mapping the stimulation position via color variation) [111]. The dual-signal sensing offers visible user interactive interface with excellent anti-interference performance, enlightening the perceivable ionic skins with bright prospects of multifunctional applications.

### 3.4. Piezoelectric

**Figure 4:**
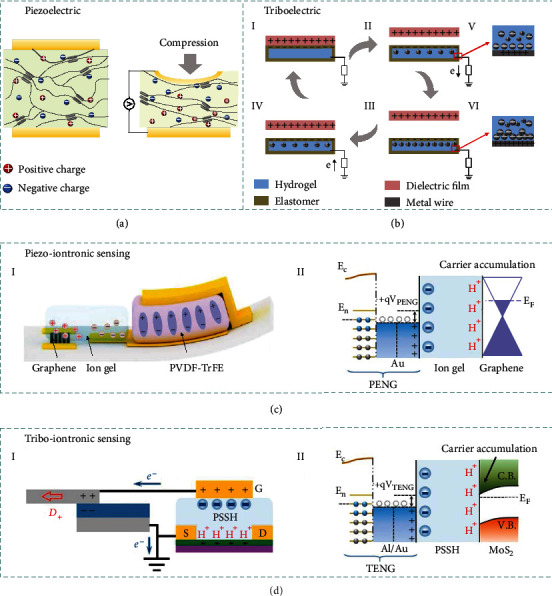
Working mechanisms of ionic sensing devices. (a) Piezoelectric [127]. Reproduced with permission, copyright 2021, Wiley-VCH GmbH. (b) Triboelectric [107]. Reproduced with permission, copyright 2017, The Authors, some rights reserved; exclusive licensee American Association for the Advancement of Science. (c) Piezoiontronic sensing [152]. Reproduced with permission, copyright 2019 WILEY-VCH Verlag GmbH & Co. KGaA, Weinheim. (d) Triboiontronic sensing [155]. Reproduced with permission, copyright 2020, American Chemical Society.

Piezoelectricity means the mechanical force induced electrical charges in certain materials derived from the occurrence of electrical dipole moments. Typically, the electrical dipole moments are originated from the deformation of noncentral symmetric crystalline structures or porous electrets with long-lasting charges in the pores [126]. Specifically, the ionic material-based piezoelectric sensor is prepared via swelling the piezoelectric polymer with electrolyte salt. The external deformation of the ionic material-based piezoelectric sensor can induce spatial distribution changes of multiple ionic constituents in the scaffold polymer networks, as depicted in Figure 4(a). The piezoelectric effect under deformation is originated from (i) the instantaneous stress distribution along the direction normal to the applied force established in the piezoelectric polymers and (ii) the different migration capabilities of the ionic constituents upon the built-in stress distribution in the polymer. The components with strong mobility will escape away from the interface where the force is applied, whereas components with weak mobility prefer to remain at the interface [127].

Derived from the classical piezoelectric theory, the surface potential (*V*_OC_) is given as [128]

(11) $$\begin{array}{c} V_{OC}=\frac{d_{ij}}{\varepsilon_{r}\varepsilon_{0}}\sigma_{ij}g_{e}, \end{array}$$

where *d*_*ij*_ is the piezoelectric coefficient and *ε*_0_ and *ε*_*r*_ are the permittivity of vacuum and relative dielectric constant, respectively. *σ*_*ij*_ represents the applied stress, and *g*_*e*_ is the gap distance between electrodes.

The management of the ion migration properties can further increase the voltage output of piezoelectric ionic materials. According to previous reports, the piezoelectric ionic materials are regarded to have thick EDL with ionic mobility (*μ*) defined by [106]

(12) $$\begin{array}{c} \mu =\frac{2\varepsilon_{r}\varepsilon_{0}a_{p}E}{3\eta }, \end{array}$$

where *η* is the dynamic viscosity of a solvent. *ε*_*r*_ represents the relative dielectric constant, *ε*_0_ is the permittivity of vacuum, respectively. *α*_*p*_ denotes atomic radius and *E* denotes elastic modulus. Most parameters (except *α*_*p*_) remain constant, indicating that cations (with the largest ionic radius) have the highest ionic mobility.

The sensitivity can be defined by

(13) $$\begin{array}{c} S=\frac{\Delta V}{\varepsilon }, \end{array}$$

where Δ*V* is the variation of potential and *ε* is the strain. Leveraging ionic materials, the ionic material-based piezoelectric sensor can reach the sensitivity up to 2.6 mV.

The piezoelectric mechanism has gradually become a simple and effective method to scavenging circumambient mechanical energy due to the advantages of low cost, straightforward fabrication process, and the capability to integrate with other sensing technologies. Furthermore, the ionic material-based piezoelectric sensor can achieve reversable and repeated deformation, which is promising for long-time use. Although the piezoelectric output signals responding to mechanical deformation are limited by the intrinsic properties of the piezoelectric materials (need to be further explored), the ionic material-based piezoelectric sensor may compensate a portion of specific applications that require high sensitivity, conformability, adaptability, spatiotemporal interaction, etc.

### 3.5. Triboelectric

The emerging TENG, which can readily convert mechanical energy into electrical energy by coupling triboelectrification and electrostatic induction, has been extensively explored to realize self-powered sensing and energy harvesting [129–135]. TENGs have four types of working modes, e.g., contact-separation mode, sliding mode, freestanding mode, and single electrode mode [136]. By replacing the friction (or electrode) layer into ionic materials, ionic TENG can be readily obtained.

Figure 4(b) illustrates corresponding working mechanism of TENG composed of ionic materials. We take the ionic TENG in single electrode as the example, in which the ionic material is grounded via a metal wire through an external load resistance. Once an object is brought into contact with the elastomer of the TENG, triboelectrification takes place at the interface, generating equal amounts of charges in opposite polarities on the surfaces of the elastomer and the dielectric film, respectively (I). The two surfaces possess the same electrical potential theoretically because the two opposite charges almost coincide on the same plane. When the dielectric film begins to separate and move away, the induced electrostatic charges on the insulating elastomer surface will lead to ion migration in the hydrogel to keep the charge equilibrium, establishing a layer of excessive ions at the interface (II). Meanwhile, an EDL is established at the metal/hydrogel interface, with equal amounts of negative ions at the interface and counter positive charges in the metal (V). With the separation distance further increased, electrons continuously flow from the metal wire to the ground until all the electrostatic charges in the elastomer film are screened (III). When the dielectric film reapproaches the elastomer film, a reversed process occurs, in which an electron flux in opposite direction will flow from ground to metal/hydrogel interface through external load resistance (IV). Periodic contact-separation process between the dielectric object and ionic TENG will induce continuous alternating currents to be generated [107].

The relationship between the output open-circuit voltage (*V*_OC_) and the transferred charges (*Q*_SC_) can be derived from

(14) $$\begin{array}{c} Q_{\text{SC}}=V_{OC}C_{0}, \end{array}$$

where *C*_0_ represents the capacitance of the TENG. In contact case, *V*_OC_ and *Q*_SC_ are both 0. In separating case, *V*_OC_ can be given by

(15) $$\begin{array}{c} V_{\text{OC}}=\frac{-\sigma A}{2C_{0}}, \end{array}$$

where *σ* denotes the electrostatic charge density generated at the surface and *A* denotes the contacting area. According to Equation (14), ultrahigh capacitance in the formed EDL will lead to great enhancement in the energy-harvesting performance for ionic TENG.

Recent studies have found that contact electrification at liquid/solid interfaces is involved with electron transfer process, which dominantly contributes to the formation of charged solid surfaces (different from traditional mechanisms of ionization, dissolution, or ion adsorption of solid surfaces) [137–142]. Based on these results, Wang's hybrid EDL model has been proposed to describe the formation process of EDLs by two steps: contact electrification and subsequent electrostatic counterion adsorption [137, 143]. According to the EDL capacitive sensing mechanism, similar strategy of structure engineering on iontronic capacitive sensors is also applicable to ionic TENG. Jia et al. introduced pyramid patterns to PAM hydrogel-based TENG to enhance the pressure sensing performance [144]. The top surface of the hydrogel pyramid forms dynamic EDL together with the PDMS, while the bottom hydrogel surface forms static EDL together with a metal wire. By designing dynamic EDL at hydrogel-dielectric elastomer interfaces, stretchable self-powered pressure sensors are constructed based on the mechano-to-electrical energy conversion mechanism. The sensitivity is optimized to be 1.40 kPa^−1^ in the low-pressure range of 31-300 Pa due to the increased elastomer roughness.

Due to high stretchability and transparency, ionic materials are widely employed in flexible TENGs as the electrodes, which are of great significance for achieving soft/stretchable self-powered systems. Besides, considering the high biocompatibility and conformability of the ionic materials, the iontronic TENGs have potential to be easily attached on human skins and even implantable inside human body. As an additional benefit, certain iontronic TENGs are capable of autonomously self-healing at room temperature [108, 145], which replicates the wound healing process of biological skins. Hence, adopting ionic conducting current collectors as the electrodes for TENG is an effective technology to improve its energy-harvesting and sensing performances. According to the formation of EDL and additional functionalities (e.g., such as stretchability, transparency, and self-healing properties), introducing ionic materials opens up new possibilities for the exploitation of TENG applications, including self-powered ionic skins, wearable/implantable electronics, low-energy-consuming healthcare monitoring, and intelligent robotics. It enables a huge step toward realizing the next-generation energy-efficient electronics.

### 3.6. Integrated Iontronic Sensors

In addition to the conventional iontronic sensing mechanisms, other novel transduction mechanisms originated from integrated systems are proposed in this review for the first time. For instance, piezo-/triboiontronics coupling EDL semiconductor devices with piezo-/triboelectric nanogenerator are emerging interdisciplinary research directions toward intelligent and high-performance sensors. Piezo-/triboiontronics are highly promising according to the following reasons. Firstly, the integrated PENGs/TENGs are desirable in low-frequency and high-entropy mechanical energy scavenging [146], micro-/nanopower package [147, 148], and driving the self-powered active systems in the era of Internet of Things [149], which offers new possibility for distributed energy harvesting and storage [150]. Secondly, iontronic semiconductor devices coupled with PENGs/TENGs have great potential for designing sophisticated device architectures that go beyond Moore's law in terms of diversity and versatility to enable human-robot interactive interface and artificial intelligence.

#### 3.6.1. Piezoiontronic Sensing

Piezoiontronics, as a kind of functional electronic devices, integrates PENG with ion gel-gated transistor, in which piezopotential is employed as gate to control or modulate the transport of charge carriers in the semiconductor channel or electrical junctions [151]. Piezotronics have shown great significances in a broad spectrum of applications, e.g., mechanical energy harvesting, electromechanical memories, strain-gated transistors and logic devices, high-resolution addressable matrix, biological sensors, biomedical therapy, and potential self-powered systems. Besides the interface modulation by piezopotential in piezotronics devices, the piezoiontronic device can be broadly defined as the PENG powered/modulated iontronic devices.

Figure 4(c) illustrates a typical architecture of piezoiontronic device. Normally, the piezoiontronic device covers a series of devices that PENG (as the driving/sensing component) is integrated to EDL transistor (as processing unit). The working process is achieved by capacitively coupling the mechanical strain-triggered piezopotential to the transistor device via the ion gel. At the beginning, the cations and anions are randomly distributed in the ion gel, keeping a state of equilibrium. Once a compressive strain imposed to the P(VDF-TrFE), a positive piezopotential will be coupled to the ion gel by the enhanced dipoles, equivalent to applying a positive gate voltage (*V*_G_) to the graphene transistor. In this case, the cations and anions of ion gel will migrate to gate/ion gel/graphene interfaces (i.e., forming EDLs), respectively (I). Since the Fermi level of graphene is shifted upward, the electrons in the channel dominate the charge transport, resulting in a decreased output current [152] (II). After the release of the compressive strain (i.e., the piezopotential vanishes), the cations/anions in ion gel will drift back to the equilibrium state. Correspondingly, tensile strain can induce a negative piezopotential, equivalent to applying negative *V*_G_ to the transistor. As the cations/anions migrate in opposite directions, the graphene Fermi level is shifted downwards and the output current is increased. It is worth mentioning that EDLs formed in ion gel-gated transistors lead to an extremely high electric field that allows efficient control of charge carriers in semiconductor channel.

Piezoiontronic sensing bridges PENG modulation and iontronic semiconductor devices, offering an active and low-power consumption method to effectively modulate semiconductor devices via external stimuli, which holds great prospects for neurorobotics, piezoiontronic logic gates, human interactive interface, and self-powered artificial intelligence [153].

#### 3.6.2. Triboiontronic Sensing

Recently, emerging triboiontronics enables efficient incorporation of TENG with ion gel-gated transistor, in which triboelectric potential is employed as gate to control or modulate the charge carrier transport in the semiconductor channel or electrical junctions. Benefiting from Maxwell's displacement current of TENG to serve as the driving force for gate modulation [154], triboiontronics provides a versatile platform for multifunctional and high-performance electronics originating from mechanical behaviors.

Figure 4(d) demonstrates the working mechanism of the triboiontronic transistor (via proton conductor) regarding to the contact-electrification-induced charge transfer, ion (proton/polyanions) distribution, and related energy band diagrams [155]. In the triboiontronic transistor, it is prepared by connecting the Al friction layer of TENG component to the transistor gate meanwhile the other electrode of TENG is connected to source electrode and grounded. Initially, there is no triboelectric potential coupling to the MoS_2_ channel without mechanical displacement from TENG component. When *D*+ (denotes opposite displacement of Al and PTFE) is imposed to the TENG, the induced positive triboelectric charges in Al film transfer to the gate of MoS_2_ FET (equivalent to applying +*V*_G_). Due to electrostatic induction, the protons will be repelled to the PSSH/MoS_2_ interface by the transferred positive charges and electron density in MoS_2_ channel increases (I). As illustrated by the relevant energy band diagram (II), a positive triboelectric potential is coupled to the MoS_2_ transistor triggered by *D*+, contributing to the proton accumulation at PSSH/MoS_2_ interface to tune the Fermi level of MoS_2_ channel. In contrast, imposing *D*− (denotes relative displacement of Al and PTFE) is equivalent to applying a negative *V*_G_. Since most protons will be attracted to the Au/PSSH interface by the transferred electrons, the electron density in MoS_2_ channel decreases and leads to upward bending of MoS_2_ energy band. Triboelectric potential gating can effectively modulate charge carrier density and energy band bending via ionic (or protonic) dielectrics.

Leveraging ion migration resulting from mechanical stimuli to modulate electronic properties, triboiontronics delivers new interdisciplinary research directions for applications in tribotronic logic gates, phototransistors, memory, tactile switches, etc. [156]

## 4. Applications

As aforementioned, iontronic sensors have considerable merits benefiting from their unique material properties, diverse sensing mechanisms, and elaborate device structure. Such distinguished advantages not only allow iontronic sensors to satisfy the requirements for wearable healthcare systems but also enable unique sensing modalities for future soft electronics, especially in artificial skin and human-machine interactive interfaces. According to different working mechanisms of iontronic sensors, in this section, typical applications are divided into six categories based on different working mechanism of iontronic sensors: (i) interfacial capacitive iontronic sensors for ordinary ionic skins and human interactive interfaces, (ii) resistive iontronic sensors for wearable healthcare monitoring and motion recognition, (iii) mechano-electroluminescent/electrochromic for visually interactive devices, (iv) piezo-/triboelectric self-powered active sensors, (v) piezo-/triboiontronic sensory transistors, and (vi) piezo-/triboiontronic artificial synapses.

### 4.1. Interfacial Capacitive Iontronic Sensors for Ordinary Ionic Skins and Human Interactive Interfaces

Ionic skins and human interactive interface applications are envisioned to be the devices that offer prompt skin-like perceiving to replicate the function of the tactile sensation of biological skins interacting with the external environment.

**Figure 5:**
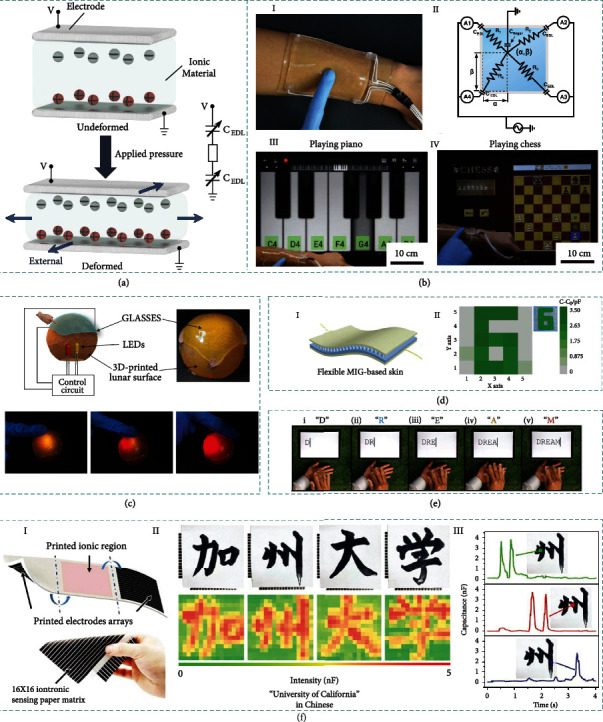
Ionic skins and human interactive interface applications of iontronic sensing. (a) The working mechanism of interfacial capacitive sensing. (b) A highly stretchable and transparent ionic touch panel capable of playing piano and chess [105]. Reproduced with permission, copyright 2016, American Association for the Advancement of Science. (c) Schematic drawing showing how a conformable pressure sensor is configured by covering a spherical “lunar” surface with a piece of GLASSES. The LED light varied between different touching areas, and its light intensity varies with the pressure [78]. Reproduced with permission, copyright 2019, The Author(s), under exclusive license to Springer Nature Limited. (d) A PDMS stamp in the shape of “6” placed on the ionic sensors array and corresponding spatial mapping of the capacitance changes [79]. Reproduced with permission, copyright 2018, WILEY-VCH Verlag GmbH & Co. KGaA, Weinheim. (e) Wireless real-time communication [65]. Reproduced with permission, copyright 2018, The Author(s). (f) The tracking and mapping of the pressure during the brush-writing process of Chinese calligraphy [158]. Reproduced with permission, copyright 2019, WILEY-VCH Verlag GmbH & Co. KGaA, Weinheim.

Interfacial capacitive sensing lies on the conversion of external force vibrations into EDL capacitance change of the sensor. The interfacial capacitive sensing has proved to be effective in recognizing stress, strain, shear, torque, and other external stimuli with high sensitivity via establishing EDL at the ionic dielectrics/electrode interface (Figure 5(a)). Besides, it can provide additional advantages of high transparency, flexibility, and biocompatibility, which is fascinating to devising ionic skins and human interactive interfaces. Therefore, numerous ionic skins and human interactive interfaces based on interfacial capacitive sensing have been reported.

Current transparent interactive touch panels all use rigid materials, but the emerging ionic skins and human interactive technologies are expected to benefit from stretchability and biocompatibility for further integration with human body. As shown in Figure 5(b), Kim et al. reported a new interactive modality of highly stretchable and transparent ionic skin based on polyacrylamide hydrogels (I) [105]. The ionic skin can be stretched extensively without influencing touch sensitivity. Utilizing a surface-capacitive system, the same voltage is applied to each corner of the ionic panel to ensure the uniform electrostatic field is imposed throughout the whole ionic panel. Once the finger touches the ionic panel, the touch site is grounded immediately, and a potential difference is induced between the touch site and electrode due to the variation of capacitance. The potential difference leads current to flow from electrode to ground. Thus, according to the touch-induced current achieved from four current meters, the touch position can be recognized based on the proportional relationship between the current and position (II). Relying on the position recognition function of the ionic skin, various motions could be successfully perceived, such as tapping, holding, dragging, and swiping. Moreover, diverse interesting interactions are explored to extend the functions of the panel, such as playing music (III) and playing chess (IV).

To further enrich the capability of the ionic skin, Cao et al. utilized reversible ion-dipole interactions to enable the self-healing function of ionic skin even in aquatic environments, which shows the potential to emulate gelatinous underwater invertebrates [78]. The self-healing ionic skin successfully implements touch position sensing based on the interfacial capacitive mechanism. Taking advantage of its high transparency and stretchability, the ionic skin is conformably attached onto a spherical surface with red and yellow LEDs mounted inside to demonstrate a human interactive interface, as shown in Figure 5(c).

In addition to material selection, microstructure processing and surface functionalization are also effective approaches to broadening the functions and improving the performance of ionic skin and human interactive interface [118, 157]. Templated from a Calathea zebrine leaf by soft lithography, Qiu et al. developed a low-cost microstructured ionic gel to obtain high-sensitivity capacitive ionic skins [79], as illustrated in Figure 5(d). The ionic skin is applied for spatial pressure identification and human-robot interaction, opening new path to adopting natural material structures to enhance the performance of ionic skin. Another work introduces self-cleaning function into ionic skin by surface functionalization of (heptadecafluoro-1,1,2,2-tetrahydrodecyl) trichlorosilane [65]. It can not only ensure the ionic skin optically and electrically stable but also improve sensitivity without sacrificing transmittance. A wireless real-time communicator based on the self-cleaning ionic skin is demonstrated in Figure 5(e). Relying on the unique advantages of papers (such as foldability, cuttability, and printability), Li et al. introduced a novel flexible iontronic sensing method to extend the iontronic sensation to more adaptive material systems by integrating the ionic material with paper (Figure 5(f)) [158]. Interestingly, the iontronic sensing paper is endowed with tactile sensation capacity, and the prepared iontronic paper can distinguish the handwriting pattern and the magnitude of applied pressure. This work is meaningful due to the successful duplication of specific calligraphy with detailed handwriting arts into a sustainable digital form. The low-cost paper-based iontronic device can be highly desirable to recognize various writing habits for accurate requirement in decryption and encryption applications as a biometric identification device.

Given the attracting merits of iontronic sensing, ionic skin and human interactive interface present a great promise in the upcoming era of artificial intelligence.

### 4.2. Resistive Iontronic Sensors for Wearable Healthcare Monitoring and Motion Recognition

Wearable healthcare monitoring and motion recognition are another hot topic that contemporary researchers are devoted to investigate [159, 160]. With extraordinary sensitivity and high conformability, the iontronic sensors are primarily expected to be capable to reconstruct the skin functions. They are also required to be wearable and capable to provide users with rich healthcare and motion-monitoring information under complicated conditions. Mechano-piezoresistive and interfacial capacitive sensing mechanisms are the most common methods to designing related wearable electronic devices. A variety of healthcare monitoring and motion recognition applications have been reported, including heart-rate monitoring, voice recognition, and noninvasive cardiovascular pressure recording.

**Figure 6:**
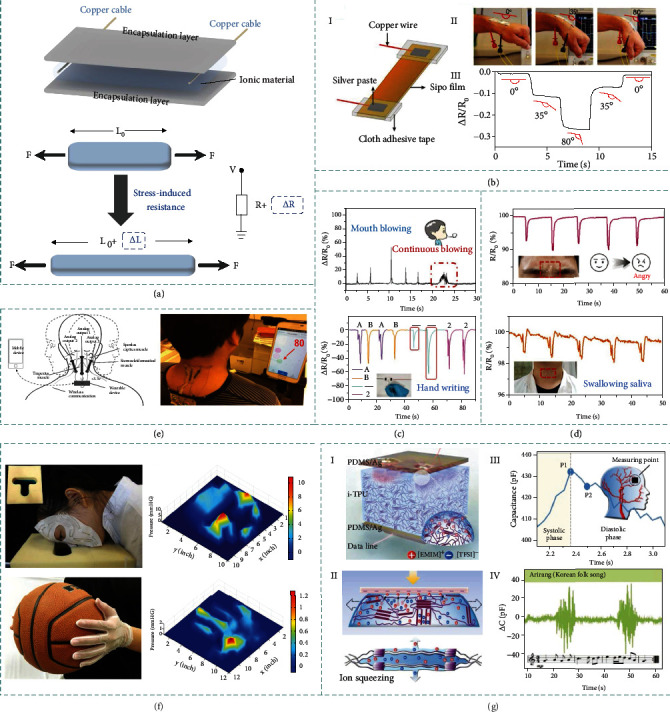
Wearable healthcare monitoring and motion recognition applications of iontronic sensing. (a) The working mechanism of mechanoresistive sensing. (b) A SiPo-based human motion detection sensor [97]. Reproduced with permission, copyright 2019, The Authors. Published by WILEY-VCH Verlag GmbH & Co. KGaA, Weinheim. (c) Relative resistance changes to mouth blowing hand writing [161]. Reproduced with permission, copyright 2020, WILEY-VCH Verlag GmbH & Co. KGaA, Weinheim. (d) The ionic-gel sensor was attached to the forehead (I), and throat (II) to detect subtle movements of the human body, such as facial expression, and swallowing saliva [162]. Reproduced with permission, copyright 2021, Wiley-VCH GmbH. (e) Strain-sensitive iontronic mechanosensor design for cervical spondylosis according to the strain directions of the skin during different neck movements and monitoring process [120]. Reproduced with permission, copyright 2016, The Royal Society of Chemistry. (f) Facial and hand pressure distribution mapping [109]. Reproduced with permission, copyright 2017, WILEY-VCH Verlag GmbH & Co. KGaA, Weinheim. (g) Design of artificial ionic mechanotransducer (I) and detailed view of the capacitive artificial ionic mechanotransducer under the stimulus (II). Recording of the radial artery pressure wave for medical diagnosis showing two distinguishable points (P1 and P2) distributed in the systolic and diastolic phase (III). Korean traditional folk music (Arirang) with frequency diversification recorded in real-time as the ultimate sound recognition (IV) [163]. Reproduced with permission, copyright 2017, WILEY-VCH Verlag GmbH & Co. KGaA, Weinheim.

According to the simple architecture and facile measurement method, mechano-piezoresistive devices are quite appropriate for such wearable applications which rely on the deformation of sensory devices. In general, mechano-piezoresistive iontronic sensor is fabricated by embedding ionic materials in encapsulation layer. Once the stress is applied, there will be a change in the cross-sectional area and length of ion-conducting channels, to induce the resistance change (Figure 6(a)). Based on protein-transformed ecofriendly ionic materials, Kadumudi et al. have developed an eco-friendly bendable iontronic sensor for human motion sensing [97]. Attaching the sensor onto the wrist, the movements of the wrist can be tested by the impedance changes, as depicted in Figure 6(b). The motion-sensing experiments are also extended to the locomotion of other major joints of human body, e.g., fingers, elbows, shoulders, knees, and ankles.

In addition to the large-amplitude motion detection, tiny and subtle movements are also critical and investigated. A smart zwitterionic conductive hydrogel is developed by Huang et al. to demonstrate good sensing properties in monitoring human motion and physiological signal response [161]. As shown in Figure 6(c), the sensing applications of subtle movements by handwriting and mouth blowing are explored. As writing different words needs different forces and writing sequences, different letters by handwriting will yield different characteristic electrical signals which can reflect unique characteristic of hand writing to be distinguished. Similarly, the induced signals are also in accordance with the changes of mouth blowing due to the different blowing frequencies and strength.

Yu and Wu designed an underwater iontronic sensor for communication and optical camouflage [162]. It can also be used for biological monitoring. For instance, by attaching it on the forehead, the sensor can detect tiny changes of facial expressions. Fixing the iontronic sensor on the throat, the process of swallowing saliva can be monitored by the sensor (Figure 6(d)). Putting it on abdomen surface, breathing modes (regular/rapid breath) and breathing process (inhaling and exhaling) can be reflected in obtained signals.

For healthcare monitoring, Wang et al. prepared a smart and highly durable wearable biomedical sensor to detect cervical movements, which has exhibited big potentials for healthcare monitoring, especially for the people suffering from cervical spondylosis (Figure 6(e)) [120]. Such device is highly desired in healthcare monitoring for office workers sedentary in front of computer. There are also wearable healthcare and motion recognition devices with high sensitivity based on the interfacial capacitive mechanism, contributed by the establishment of EDL.

Monitoring facial pressure distribution is essential to plastic surgery or related clinical situations, a supercapacitive iontronic nanofabric facemask embedded with sensors has been successfully designed to map the facial pressure distribution, as shown in Figure 6(f). The reported iontronic nanofabric facemask can be employed to monitor facial pressure distribution for plastic surgery. It can provide prompt feedback and adjust the head position in real time over prolonged surgery procedures to avoid excessive pressure imposed by head and body weights [109]. Additionally, the hand pressure distribution is demonstrated to deliver delicate and subtle action guidance for the basketball players. When one grips a basketball with sensory gloves, corresponding pressure distribution with detailed gripping force information can be efficiently resolved in high-resolution and real-time. As an additional benefit, the conventional woven silk substrate supplies the glove with excellent gas permeability and water excretion when contacting skin to prevent ulcer. Extensively, such sensor can be applied to measure blood and pulse pressure [80].

The iontronic sensor can be further extended to versatile applications, e.g., preventing an early cardiovascular problem. As demonstrated in Figure 6(g), Jin et al. fabricated a capacitive artificial ionic mechanotransducer with ion nanochannels (I), which possesses the property overpassing the Merkel cell detection [163]. The mechanotransducer is capable of perceiving a very wide range of pressures (10 Pa to 50 kPa) in the human-adaptive tactile systems, which is much broader than the light touch perceived by human's Merkel cells. Being subjected to even subtle stress, the ions are extruded from the free volume of the i-TPU matrix, thus breaking the ion equilibrium (II). For healthcare diagnosis, a radial artery pulse is detected with the sensor attached to the forehead, targeting at people suffering from cardiovascular disease who requires a high-fidelity recording to accomplish daily diagnosis. The systolic and diastolic phases correspond to the highlighted points (P1 and P2) (III). Moreover, this iontronic sensor is explored to detect acoustic pressure with a high-pressure resolution (10 Pa at 3-4 atm) (IV).

Based on above discussions, the iontronic sensors are undoubtedly broadening their applications in wearable medical devices and motion recognition, suggesting a great potential for intelligent healthcare monitoring, recovery, and corrective exercises.

### 4.3. Mechano-Electroluminescent/Electrochromic for Visually Interactive Devices

**Figure 7:**
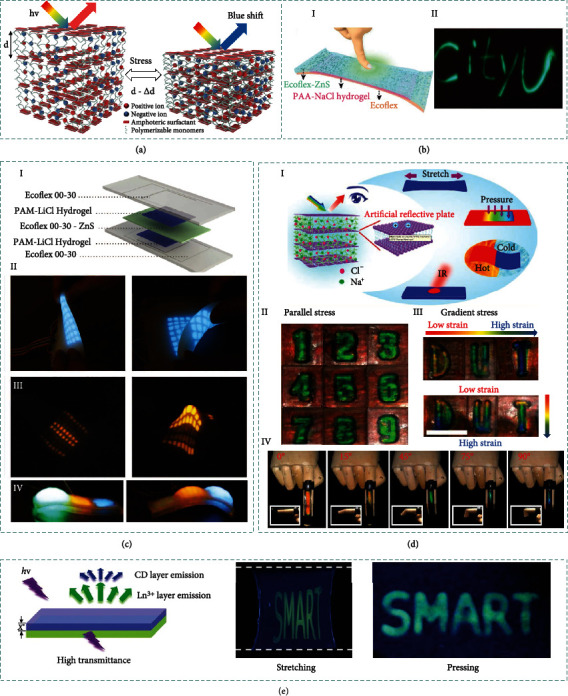
Visual interactive displays applications of iontronic sensing. (a) The working mechanism of mechano-electrochromic sensing. (b) Structural configuration of the smart skin device and image resolution of the written electroluminescent letters as “CityU” [112]. Reproduced with permission, copyright 2019, WILEY-VCH Verlag GmbH & Co. KGaA, Weinheim. (c) Highly stretchable user interactive display. Exploded view of the hyperelastic light-emitting capacitor showing its five-layer structure consisting of a ~1 mm thick electroluminescent layer (ZnS-Ecoflex 00-30) that is sandwiched between two PAM-LiCl hydrogel electrodes and encapsulated in Ecoflex 00-30 (I). Multipixel electroluminescent displays fabricated via replica molding (II). With subsets of pixels activated, subsets of pixels are activated while being deformed (III). An undulating gait is produced by pressurizing the chambers in sequence along the length of the crawler (IV) [113]. Reproduced with permission, copyright 2016, American Association for the Advancement of Science. (d) Illustration of a biomimetic skin with multifunctional visual sensing (I) and photographs showing the color changes induced by the convex letters superimposed with a uniform (II) and a gradient (III) stress field [111]. The biomimetic skin attached to a finger under stepwise bending (IV). Reproduced with permission, copyright 2021, American Chemical Society. (e) Design principle and applications of double-layer mechanochromic materials based on the strain-transmittance relationship [164]. Reproduced with permission, copyright 2019, WILEY-VCH Verlag GmbH & Co. KGaA, Weinheim.

Stretchable and flexible display devices are important components in visible user interactive interface, enlightening the multifunctional perceiving ionic skins with bright application prospects. Mechano-electroluminescent and mechano-electrochromic mechanisms are two main principles in designing dual-signal sensing devices. Mechano-electroluminescence transduces the mechanical pressure into a light emission, while mechano-electrochromic responds to the stimuli with color change (Figure 7(a)).

Taking advantage of soft and stretchable ionic materials, the iontronic sensors are possible to provide a completely stretchable platform for human-machine interfaces. Mechano-electroluminescent additives are embedded into the stretchable ionic materials, endowing the iontronic sensors with light emission responses to mechanical stimuli in real time. The combined properties excited new applications of iontronic sensor in user-immersive displays. Leveraging mechano-electroluminescence effect, Liang et al. reported a dual-modal iontronic sensor in which stretchable hydrogel electrodes are sandwiched between Ecoflex layer and mechanoluminescent layer embedded with ZnS phosphor (Figure 7(b)). The mechanical stability is ensured by an in situ assemble method (I) [112]. The light-emitting response is experimented to enable visualization of both the magnitude and site of the applied force (II). Notably, even when being subjected to a strain up to 700%, the as-developed iontronic sensor exhibits extraordinary vitality without reduction in functionality.

Larson et al. utilized PAM-LiCl hydrogel as stretchable electrodes to devise a light-emitting capacitive iontronic sensor (Figure 7(c)), integrating perceiving of external mechanical stimuli with a visual response [113]. The five-layer architecture is constituted by ZnS phosphor-doped silicon dielectric layer sandwiched between two hydrogel electrodes and then encapsulated in low Young's modulus Ecoflex (I). This design is to ensure high sensitivities in pressure sensing by capacitance and illuminance. The light-emitting display enables the anti-interference user interactive visual response even undergoing different types of deformation (e.g., stretching, wrapping, rolling, and folding) (II). The electroluminescent device can be additionally extended to multipixel electroluminescent display and multitouch sensing at a subcentimeter scale resolution activated by deformation (III). Interestingly, assembling such highly stretchable and transparent displays into soft actuators boosts two additional advantages in soft electronics, i.e., actively shape-adjustable displays and illuminance-modifiable soft robots.

Mechano-electrochromic sensing is another important modality of sensing system with dynamic color variation and prompt sensory feedback, which allows for versatile applications, such as visualized dynamic displays, multifunctional interactive sensing, and intuitive intelligent interactions with users [114]. Based on mechano-electrochromic sensing, Wang et al. demonstrated an intelligent chromotropic iontronic device by constructing bio-inspired ultrastructures with anisotropic electrostatic repulsion functions [111]. As displayed in Figure 7(d), benefiting from adjustable geometric structures as well as ionic mechanotransduction mechanism, the chromotropic iontronics can achieve optical/electrical feedbacks to mechanical pressure synchronously (I). Notably, digital “1−9” patterns present almost the same color when being subjected to uniform stresses (II). Yet a blue-shift phenomenon is observed when the intelligent chromotropic iontronic device is compressed under gradient stress (III). Furthermore, the device is explored to monitor the bending motion of finger. When the finger bends stepwise, the reflection wavelength decreases and induces the blue-shift of structural color. Meanwhile, the relative resistance signal shows a stepwise increment, suggesting that the biomimic ionic skin can recognize finger bending precisely (IV).

To further enrich the functions of the visual interactive interfaces, Zhu et al. developed a novel double-layer luminescent electrochromic hydrogels with embedded two luminescent species (i.e., carbon dots and lanthanide ions), which can realize the function of encryption and decryption [164]. The mechano-electrochromic sensing is realized by adjustable transmittance of the top layer dependent on applied strain, which controls the overall luminescence by tuning light emission from the bottom layer, as illustrated in Figure 7(e). As a proof of concept, by encoding confidential information into the bottom layer, an encryption device is developed which can only be decoded by mechanical cues.

Remarkably, being integrated into soft robots, the iontronic visual interactive interface can functionalize as camouflage equipment with dynamic color variation and sensory feedback, which further enriches the functions of the soft electronics. All the characteristics of iontronic sensor (e.g., transparency, stretchability, and compatibility) promise a bright future in the fields of visually interactive technologies.

### 4.4. Ionic PENG/TENG for Self-Powered Active Sensors

Emerging soft electronics and bioelectronics have propelled immense enthusiasm for the exploitation of diverse electronic devices with transparency, deformability, and self-healing ability [108]. Although rapid progress has been achieved, the power supply remains challenging for sustainable electronic devices. The development of PENG and TENG has brought new vitality to self-powered soft electronics.

**Figure 8:**
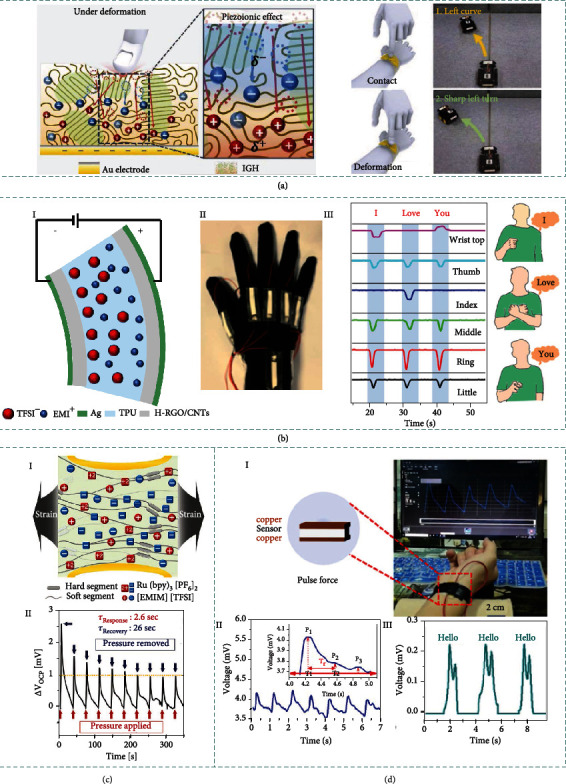
Self-powered active sensors of iontronic sensing. (a) Schematic illustration of ion dynamics induced by the piezoelectric effect and wearable dynamic sensing-based wireless communication system [106]. Reproduced with permission, copyright 2021, Wiley-VCH GmbH. (b) Working mechanism and sign recognition of the ionic sensor with smart glove [166]. Reproduced with permission, copyright 2017, American Chemical Society. (c) Distribution of ionic components in smart skin under stretching (I). Open-circuit potential of an ionic TPU film (80 wt%) recorded under cycles of pressure (10 kPa) application and removal (II) [127]. Reproduced with permission, copyright 2021, Wiley-VCH GmbH. (d) Photograph and model illustration of a wearable pulse monitor for a volunteer to measure the radial artery (I). A pulse waveform of a healthy man (II). Voltage signal output diagrams for speaking words (III) [167]. Reproduced with permission, copyright 2019, American Chemical Society.

PENG-based self-powered iontronic sensors have been widely developed for applications in touch sensation, hand gesture recognition, etc. As demonstrated in Figure 8(a), when a deformative stimulus is imposed, ion redistribution will be induced due to the piezoelectric effect. Instantaneous ion polarization takes place as the result of ion mobility difference between cations and anions. Yoon et al. recently reported an ion-doped gelatin hydrogel which can identify the touch information with an object and the deformation state under a certain level of force [106]. Based on this device, self-powered dynamic communication is demonstrated with real-time motion feedback. Such devices have potential in achieving dynamic sensors which allow simultaneous identification of pressure and textures via ion-dynamics derived in-depth analysis [165].

Zhao et al. designed a self-powered space-discriminative iontronic sensor (Figure 8(b)) [166], which is capable to distinguish deformations in different directions with remarkable spatial resolutions. Furthermore, an iontronic self-powered intelligent glove is fabricated by Lee et al., demonstrating a multidimensional and space-discriminative detection of sign languages. Through attaching sensor arrays on hand joints, different sign languages can be distinguished via analysis of the detected output electrical signals. Lee et al. devised a piezoluminescent iontronic device that exhibited a visco-poroelastic response to mechanical pressure due to the change of inside-ion distribution [127]. When the sensor is subjected to pressure, positive and negative charge spaces will be built, which is indicated by the open-circuit voltage values upon varied pressures. Combining mechano-electroluminescent mechanism, the device is further endowed with light-emitting function, which can be used for spatial recognition by corresponding light emissions (not necessary to use conventional distributed sensory arrays for pixel recognition) (Figure 8(c)). Additionally, the piezoelectric iontronic sensors are proved to have capability to precisely detect the physiological signals and spoken words (auditory sense application, Figure 8(d)) [167].

**Figure 9:**
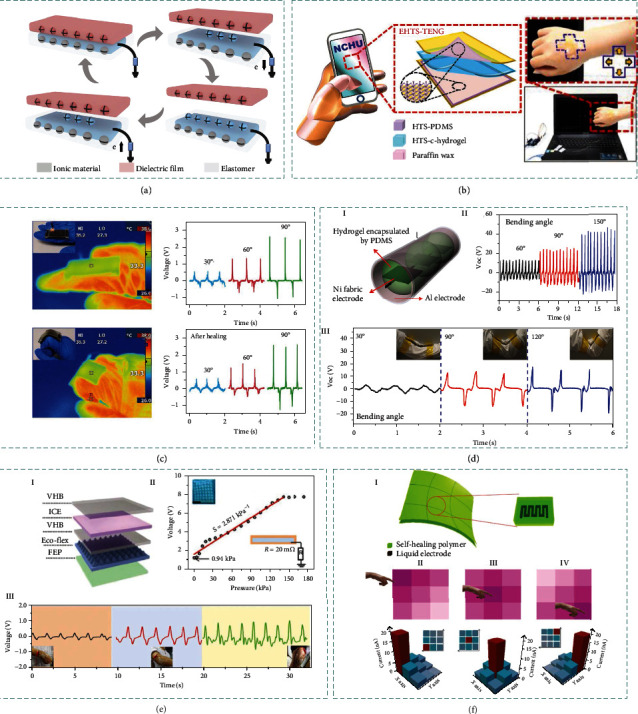
Self-powered active sensors of iontronic sensing. (a) The working mechanism of triboelectric sensing. (b) Applications and schematic of an exploded view of the entirely, intrinsically, and autonomously self-healable, highly transparent, and superstretchable TENG [168]. Reproduced with permission, copyright 2019, WILEY-VCH Verlag GmbH & Co. KGaA, Weinheim. (c) Thermal images with inset optical images of the original and bent TENG on the finger and open-circuit voltage responses when bending the finger joint at different angles (30°, 60°, and 120°) of the original and self-healed TENG [169]. Reproduced with permission, copyright 2020, WILEY-VCH Verlag GmbH & Co. KGaA, Weinheim. (d) Applications of the Hydrogel-TENG to detect joint motion [170]. Reproduced with permission, copyright 2016, WILEY-VCH Verlag GmbH & Co. KGaA, Weinheim. (e) Scheme and human activity monitoring of the ion-conducting elastomer-TENG sensor [85]. Reproduced with permission, copyright 2020 WILEY-VCH Verlag GmbH & Co. KGaA, Weinheim. (f) The multistage sensation matrix composed of the fully organic self-powered ionic skin based on TENG [171]. Reproduced with permission, copyright 2021, Lijuan Song et al. Exclusive Licensee Science and Technology Review Publishing House. Distributed under a Creative Commons Attribution License (CC BY 4.0).

TENG, with unique merits (e.g., low cost, wide material availability, easy fabrication, and diversified designability), is another efficient component as an eco-friendly and sustainable power source for various sensors. Derived from the triboelectrification and electrostatic induction between two different materials, TENG provides an effective method for mechanical energy scavenging and self-powered sensing (Figure 9(a)). Based on triboelectrification mechanism, Lai et al. fabricated a self-healable, transparent, and stretchable iontronic TENG based on HTS-c-hydrogel as the flexible electrodes (Figure 9(b)). The TENG can not only act as sustainable power source but also behave as self-powered active sensors. Furthermore, the HTS-c-hydrogel-TENG-based active ionic skin has been demonstrated in comprehensive human interactive applications, including smart glass, cellphone panel, and epidermal controller [168]. Dai et al. designed an entirely self-healing, stretchable, and tailorable iontronic TENG to monitor human motions. Remarkably, the TENG utilizes the natural infrared radiation from human skin to stimulate the self-healing process. Finger bending detection by the original and self-healed TENGs is demonstrated in Figure 9(c), which confirms the good reliability of self-healable TENG sensor [169]. Besides, the self-powered iontronic TENG sensor has also been applied to waist, elbow, knee, and other joints for corresponding motion detections. Xu et al. proposed an eco-friendly and recyclable hydrogel-TENG to harvest surrounding mechanical energy, simultaneously acting as self-powered wearable sensors (Figure 9(d)) [170]. Zhang et al. demonstrated a highly stretchable TENG with ionic conductor electrodes (ion-conducting elastomer) to monitor the human activity (the self-powered sensors are attached to waist, elbow, and knee, respectively) (Figure 9(e)) [85]. Utilizing [OMIm][PF6], Song et al. developed a self-powered skin based on TENG which can not only detect the arm swing but also perceive the motion trajectory in multistage fashion via a 3 × 3 sensation matrix (Figure 9(f)) [171].

Derived from the extraordinary properties of PENG and TENG, assisted with in-depth analysis of electronic signals, the self-powered active iontronic sensors promise as competitive candidates for future energy devices, robotics, and prosthetic skin.

### 4.5. Piezo-/Triboiontronic Sensors

Strong polarization of ionic materials can be obtained by the reverse motions of cations/anions upon applied electric field. Ionic materials, especially ionic gels, are demonstrated as a new class of highly capacitive gate dielectrics in soft EDL transistors [172, 173]. However, attributed to less structural designability and poor stability, directly employing conventional EDL transistors as sensors is restricted.

**Figure 10:**
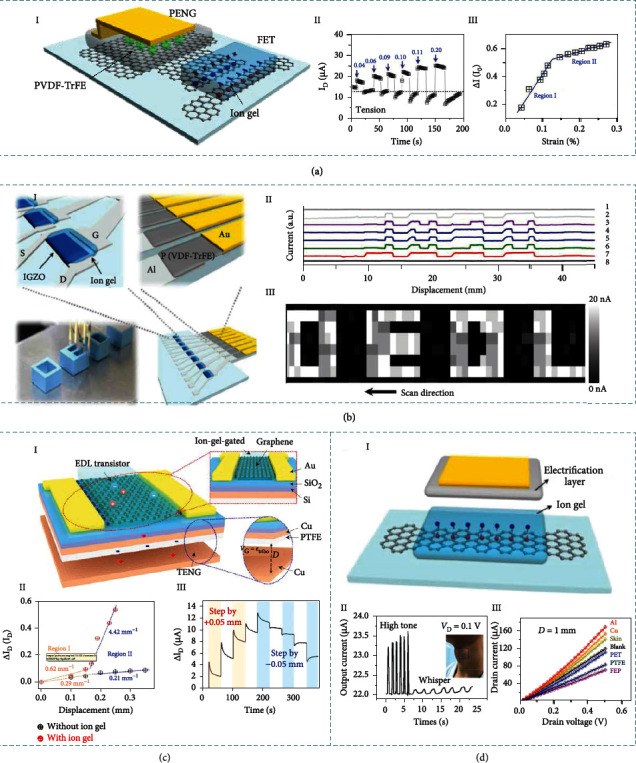
Flexible electric double-layer transistors of iontronic sensing. (a) Mechanism of graphene transistor strain sensing based on piezoelectric potential gating and sensitivity and stability characteristics of piezoelectric gated graphene transistor strain sensor [151]. Reproduced with permission, copyright 2015, WILEY-VCH Verlag GmbH & Co. KGaA, Weinheim. (b) Process for manufacturing multilevel nonvolatile memory array for piezoelectric potential programming and sensing performances of the piezopotential-programmed memory array using the target alphabet pattern “OEDL” [174]. Reproduced with permission, copyright 2016, American Chemical Society. (c) Schematic illustration of the dual-mode FET, sensitivity of the distance sensor, and real-time distance sensing test [175]. Reproduced with permission, copyright 2020, American Chemical Society. (d) Schematic illustration of the triboelectric GFET with ion gel as friction layer (I). Sensations of a high note and a whisper with the triboiontronic GFET sensor (II). Output characteristics of tribotronic GFET contact with different friction materials (separation distance is 1 mm) (III) [176]. Reproduced with permission, copyright 2018, American Chemical Society.

Derived from the advantages of high capacitance, low operation voltage, and low-power consumption, piezoiontronic sensing is extensively adopted in flexible EDL transistors. The long-range polarization feature of ionic materials permits the coplanar design of gate-to-channel. Sun et al. firstly demonstrated a piezoiontronic active strain sensor array by incorporating P(VDF-TrFE)-based PENG with all graphene transistor [151], as shown in Figure 10(a). Employing the ion gel dielectrics, the piezoelectric potential can be effectively coupled to the graphene channel, which allows the output sensory signal to maintain under the continuously applied strains. Notably, the resulting piezoiontronic sensor presents extraordinary sensitivity with a GF of 389, ultrahigh detection limit of 0.008%, and excellent mechanical durability. They further exploit a piezopotential-programmed multilevel nonvolatile memory array by incorporating ion gel-gated field-effect transistor and PENG (Figure 10(b)) [174]. The piezoiontronic memory device can be programmed through imposing external stain to the PENG. By contrast, the stored data (which is also the external mechanical information) can be erased through grounding the top electrode of the PENG. Being subjected to different external bending strains, the memory device successfully achieves multilevel data storage with superior memory performance (e.g., multilevel data storage of 2 bits, over 4 levels), great programming/erasing current ratio (>10^3^), excellent data retention over 3000 s, and reliable stability over 100 cycles. Furthermore, the real-time sensing performances of the piezoiontronic memory are investigated to simulate a target alphabet pattern “OEDL” (II and III).

As another important emerging technology, the coupling between TENG and EDL transistor leads to a new field in flexible self-powered sensors, namely, triboiontronics [156]. By virtue of triboiontronic sensing, Zhang et al. reported a dual-mode EDL transistor (i.e., a tribotronic graphene transistor with capacitively coupled ion gel-gated dielectrics) [175], as illustrated in Figure 10(c). The resultant triboiontronic gating performances are substantially improved with a twofold increase in on-state current and a fourfold increase in on/off ratio (the first mode). Moreover, the triboelectric potential determined by the displacement of TENG can efficiently modulate the output current and threshold voltage. Thus, a multiparameter distance sensor is demonstrated with a drain current increment of ∼600 *μ*A and a threshold voltage shift of ∼0.8 V when the mechanical displacement of the TENG is at 0.25 mm (the second mode). Meng et al. further extended the sensing function of triboiontronics by developing a mechanical sensory matrix with direct-contact tribotronic graphene transistors. Such triboiontronic EDL devices are competent to perceive approaching distances, classify different materials, and even distinguish voices [176].

The three-term coupling (piezoelectricity/triboelectricity, electrolyte, and semiconductor) is of considerable significance to employ EDL modulation to rapidly advance the interdisciplinary concepts of piezoiontronics and triboiontronics.

### 4.6. Artificial Synapses

Neuromorphic devices, particularly synaptic transistors, are emerging research hotspots these days. In order to fully mimic the functions of human brain, great efforts have been made to electronics capable of parallel data processing and weights updating via software or algorithms. However, the huge energy consumption remains to be a problem. Given the demands for low-power-consuming neuromorphic devices, energy-autonomous sensory neurons are highly favorable for the development of revolutionary neuromorphic systems.

**Figure 11:**
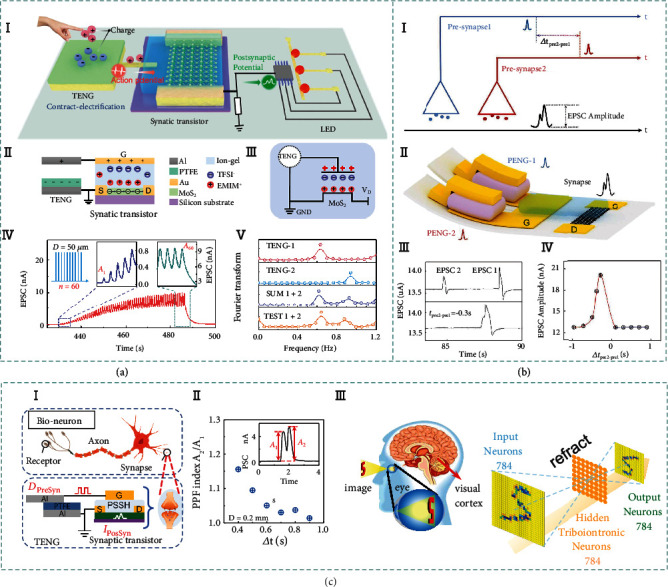
Artificial synapse of iontronic sensing. (a) Schematic illustration of the CE-activated artificial afferent. It includes a self-activation component, a synaptic transistor, and a functional circuit (I). Cross-sectional view of the CE-activated MoS2 synaptic transistor and illustration for each component (II). Circuit diagram of the CE-activated artificial afferents (III). The EPSC responses under 60 CS actions (IV). The extracted frequencies of the TENG-1 and TENG-2 activations after Fourier transform (V) [177]. Reproduced with permission, copyright 2021, The Author(s). (b) The spatiotemporal strain pulses (two tensile strains) correlated with the piezoiontronic artificial sensory synapse. Scheme of EPSC triggered by two spatiotemporally correlated pulses applied through presynapse-1 and presynapse-2 (I). Schematic illustration of the artificial neural network with two artificial presynaptic sensor neurons (PENG-1 and PENG-2) to input and one artificial postsynapse to process (II). The top panel shows the EPSC-1 and EPSC-2 triggered by strain-1 and strain-2 from the PENG-1 and PENG-2, respectively (III). The bottom panel shows that the amplitude in EPSC is the sum of EPSC-1 and EPSC-2 at tpre2 − pre1 = −0.3 s. The EPSC amplitude at *t* = 0 is plotted as a function of *Δ*tpre2–pre1 (IV) [152]. Reproduced with permission, copyright 2019, WILEY-VCH Verlag GmbH & Co. KGaA, Weinheim. (c) Artificial synaptic simulation based on the triboiontronic transistor via proton conductor [155]. Reproduced with permission, copyright 2020, American Chemical Society.

Our group demonstrated the first contact-electrification-activated artificial afferent [177]. As depicted in Figure 11(a), according to triboelectrification, the induced triboelectric potential can effectively activate the EDL MoS_2_ synaptic transistor, which endows the artificial afferent with superior property to perform spatiotemporal recognition of various external stimuli (e.g., displacements/pressures/touch patterns corresponding to I/II/III). The contact-electrification-activated artificial afferent with mechanoplastic characteristics [178] has been investigated to establish dynamic logic and recognize the frequencies and magnitudes of external actions. Taking advantage of triboelectricity, energy dissipation of the artificial afferent can be greatly reduced to 11.9 fJ per spike. Additionally, the recognition of spatiotemporal touch patterns is realized on flexible substrate. Such low-power electronics capable of spatiotemporal recognition provide a new insight into constructing self-powered bioelectronics, intelligent interactive interfaces, and neuromorphic sensory networks. Based on piezoiontronic sensing mechanism, Chen et al. developed the piezoiontronic counterpart of an artificial sensory synapse by coupling P(VDF-TrFE)-based PENG and EDL transistor [152]. The piezopotential derived from PENG can effectively power and modulate the synaptic device resulting from the EDL established at the ion gel/electrode interface and ion gel/graphene interface. Moreover, the piezopotential coupling allows for the recognition of spatiotemporal strain information with postsynaptic currents. This work is of great significance for brain-inspired neuromorphic computation based on piezoiontronic synaptic devices.

Benefitting from ionic migration (derived from the mechanical displacement) and its inherent relaxation behavior, the artificial synapses can be further explored to simulate an artificial sensory neuron system. Utilizing the triboiontronic MoS_2_ device, Yang et al. simulated a visual imaging system assisted with mechanical behavior. The visual imaging system achieves the inverted transformation of an image via a multilayer neuron simulation network, in which the synaptic weight (channel conductance) of PSSH-gated transistor is effectively updated by mechanical displacement [155], as demonstrated in Figure 11(c). Based on all-rubber materials, John et al. reported a rubber synaptic transistor which successfully realized synaptic behaviors, e.g., synaptic filtering, pair-pulse facilitation, and current enhancement after excitation. The rubber synaptic transistor further integrates TENG (as power supplies) with neuromorphic memtransistor to establish a self-healing soft adaptive neural robot [179]. The designed soft neurorobotic system is capable to perceive external mechanical stimuli and perform corresponding bending movements, which is applicable for building intelligent humanoid robots in the future.

Counting on the low-power consumption and diverse modalities of mechanosensing, the above mechanosensory artificial synapses lay down a novel framework for building an interactive nervous system with great significances for intelligent robotics and prostheses [180, 181].

## 5. Conclusions and Prospects

In this review, we highlight the advanced technologies and diversified applications for the exploitation of iontronic sensors toward ionic skins and artificial intelligence. Contributing to the ion conduction similar with biological system, iontronic sensing can not only emulate the mechanical sensation function in human skin but also extend the sensing mechanism with ion migrations under mechanical stimuli, promising iontronic mechanosensation to establish more intelligent sensing interface. Owing to the excellent stretchability, high transparency, and mechanical conformality, attempts have been made to explore various novel ionic materials to fabricate the iontronic sensors with skin-like perceptive properties, such as self-healing and multimodal sensing. Moreover, to achieve multifunctional artificial skins and intelligent devices, various mechanisms based on iontronics have been investigated to satisfy multiple functions and human interactive experience. The iontronics can be endowed with the competence of dual-signal sensing, which offer visible user interactive interface through mechano-electroluminescent/electrochromic method. Furthermore, with the advent of self-powered technologies (i.e., piezoelectric and triboelectric technologies), new opportunities for self-powered soft/wearable electronics have been introduced. By virtue of the unique material property, diverse sensing mechanisms, and elaborate device structure, iontronic sensors have enlightened the multifunctional perceiving intelligent artificial skins with bright application prospects.

Following are several prospects of the iontronic sensing for further technological exploration and possible applications. First, since ionic materials are ubiquitous in nature, the natural ionic materials can be utilized in iontronic device without complicated design, which may eliminate the challenge in biocompatibility of iontronic devices. Utilizing the intrinsic biocompatibility of natural ionic materials, future research is expected to promote the development of iontronic sensors toward medical and healthcare directions in wearable, implantable, and even digestible forms.

Second, despite self-powered technologies have alleviated the energy crisis in sensing networks, few researches have ever reported the miniaturized iontronic self-powered devices, which may limit their application for fine sensing and implantable monitoring. Future self-powered iontronic sensors are expected to miniaturize the structure and achieve fine array sensing.

Third, given the high level of the transparency, iontronic devices can be further incorporated with widely used optical components (e.g., consumer displays or medical endoscopies), which will introduce more interactive experiences to human interactive interface with fine tactile input information.

Last but not the least, the future research of the iontronic sensing technology can be envisioned to utilize sustainable ionic material to design intelligent ionic skins and even neuromorphic systems which are capable of perceiving, analyzing, feedbacking, and learning. Relying on the dramatically developing artificial intelligence, the iontronic sensors have potential to be endowed with analyzing, recognizing, and learning ability, opening a new era of intelligent iontronics. Given the growing demand on intelligent Internet of Things, iontronic sensors are believed to offer more sophisticated properties and benefits toward industrial, commercial, and medical consumers. Derived from the fascinating merits, iontronic sensors are bound to have a hunting ground in next-generation ionic skin and artificial intelligence.

## Figures and Tables

**Table 1:** Summary of materials, working mechanisms, and application of recently reported iontronic sensors.

Material type	Compositions	Stretchability	Transmittance	Ionic conductivity	Working mechanism	Sensing range	Sensitivity	Response time	Characteristics	Applications
Ionic liquids [49]	[EMIM][TCM]	—	80%	18 mS cm^−1^	Capacitive	4.2 N	29.8 nF N^−1^	12 ms	Three-dimensional force measurement	Human-machine interface
Ionic liquids [43]	NaCl, glycerol	—	—	—	Capacitive	1.56 N	7.20 pF N^−1^	0.46 s	Remote tactile sensing	Human-machine interface
Ionic liquids [48]	[EMIM][TCM]	—	—	18 mS cm^−1^	Capacitive	—	0.43 nF kPa^−1^	Several milliseconds	Flexible ultrasensitive tactile sensing	Ionic skin, healthcare monitoring
Hydrogels [105]	LiCl, PAAm	1000%	98%	1 S m^−1^	Capacitive	12 kPa	—	—	Highly stretchable, transparent ionic touch panel	Ionic skin, human-machine interface
Hydrogels [65]	LiCl, AAm	330%	99.60%	—	Triboelectric	238.8 kPa	—	—	Transparent and self-cleanable ionic communicators	Human-machine interface
Hydrogels [106]	Gelatin	—	—	42.28 *μ*S m^−1^	Piezoelectric	—	—	—	Dynamic mechanical stimuli perception	Ionic skin, human-machine interface
Hydrogels [53]	AA, MEA, CMC, Al^3+^, DMSO	694%	100%	—	Mechanoresistive	60.9 kPa	3.60 (GF)	400 ms	Dynamic pattern behavior	Ionic skin
Hydrogels [68]	Py, GAGAGY	7700%	—	—	Capacitive	20 kPa	0.2 nF kPa^−1^	A few seconds	Stretchable and self-healable hydrogel artificial skin	Ionic skin, motion recognition
Hydrogels [107]	LiCl, PAAm	1160%	96.20%	—	Triboelectric	446.2 kPa	0.013 kPa^−1^	—	Biomechanical energy harvesting and tactile sensing	Ionic skin, motion recognition
Ionic gels [80]	[EMIM][TCM], PEGDA, HOMPP	—	99%	18 mS cm^−1^	Capacitive	18 kPa	1.32 nF kPa^−1^	Submillisecond range	Flexible transparent iontronic film	Ionic skin, healthcare monitoring
Ionic gels [78]	[EMIM][TCM], PVDF-co-HFP-5545	1200%	98%	10^−3^ S cm^−1^	Capacitive	0.21 MPa	—	—	Self-healing electronic skins for aquatic environments	Ionic skin, human-machine interface
Ionic gels [108]	Na_2_B_4_O_7_, PVA	700%	92%	2.9 × 10^−5^ S cm^−1^	Triboelectric	—	—	—	Self-healing ionic skin triboelectric nanogenerators	Ionic skin
Ionic gels [70]	[EMI][ES], polyTA/ethanol gel	2000%	95%	20.3 mS m^−1^	Mechanoresistive	—	—	—	Stretchable and healable electronics	Ionic skin
Ionic gels [109]	[EMIM][TFSI], P(VDFHFP)	—	—	—	Capacitive	115 kPa	54.31 kPa^−1^	4.2 ms	Low cost, microstructured, ultrahigh sensitivity	Ionic skin
Ionic gels [110]	([EMIm][TFSI], PEGPEA	55%	—	3.55 × 10^−1^ mS cm^−1^	Mechanoresistive/mechano-electrochromic	600 kPa	1 (GF), 1.76 nm%^−1^	240 ms	Interactively visual ionic skin with optical and electrical synergy	Ionic skin, optoelectronic devices
Polyelectrolytes [85]	LiTFSI, PEGDA, BA monomer	1036%	91.5%	4.7 × 10^−6^ S cm^−1^	Triboelectric	340 kPa	2.87 kPa^−1^	—	Solvent-free ion conducting elastomer	Ionic skin, motion recognition
Polyelectrolytes [86]	[PBVIM][TFSI], PAN	—	—	—	Capacitive	10 kPa	0.49 kPa^−1^	30 ms	Washable and moisture proof pressure sensing	Ionic skin, healthcare monitoring
Ionic composites [54]	ZnSO_4_, PVA, Mxene	180%	—	56 mS m^–1^	Mechanoresistive	875 kPa	5.82 (GF)	250 ms	Wearable flexible sensors and arrays	Ionic skin, human-machine interface
Ionic composites [92]	[EMIM][TFSI], PU, sponge	—	—	—	Capacitive	118 kPa	5.28 nF kPa^−1^	—	Ionic liquid-polyurethane sponge capacitive pressure sensor	Ionic skin, healthcare monitoring
Ionic composites [90]	[EMIM][TFSI], TPU, silica	800%	—	—	Capacitive	135 kPa	5.77 kPa^−1^	60 ms	Bioinspired ultrasensitive ionic mechanoreceptor skin	Ionic skin, human-machine interface
Ionic composites [111]	NaCl, PDAAM-co-PAAM, Dyes	250%	—	1.88 S m^−1^	Mechanoresistive/mechano-electrochromic	1.7 kPa	1.4 (GF), 1.9 nm%^−1^	0.1 s	Dynamic display, multifunctional sensing	Ionic skin, optoelectronic devices
Ionic composites [112]	NaCl, PAA, ZnS	1100%	96.90%	—	Triboelectric/mechano-electroluminescent	51 kPa	0.23 kPa^−1^	—	Touch-sensing, and exteroception-visualizing	Ionic skin, optoelectronic devices
Ionic composites [113]	LiCl, PAM, ZnS	480%	—	10 S m^−1^	Capacitive/mechano-electroluminescent	30.9 kPa		—	Optical signaling and tactile sensing	Ionic skin, optoelectronic devices
Natural ionic materials [97]	Silk–laponite	—	85%	4.6 × 10^−3^ S cm^−1^	Mechanoresistive	—	—	—	Protein-based, water-insoluble and bendable	Ionic skin, motion recognition
Natural ionic materials [114]	Hydroxypropyl cellulose, PACA, CNTs	—	—	—	Mechanoresistive/mechano-electrochromic	20 kPa	—	—	Bioinspired conductive cellulose material	Ionic skin, optoelectronic devices
Natural ionic materials [99]	Cellulose, lignin, PAM, FeCl_3_	50%	—	—	Mechanoresistive	60 kPa	0.6 MPa^−1^	—	Catechol-containing-based chemistry, all-wood hydrogel	Ionic skin, healthcare monitoring
